# *Plasmodium falciparum* specific helicase 2 is a dual, bipolar helicase and is crucial for parasite growth

**DOI:** 10.1038/s41598-018-38032-1

**Published:** 2019-02-06

**Authors:** Manish Chauhan, Renu Tuteja

**Affiliations:** Parasite Biology Group, ICGEB, P. O. Box 10504, Aruna Asaf Ali Marg, New Delhi, 110067 India

## Abstract

Human malaria infection is a major challenge across the globe and is responsible for millions of deaths annually. Rapidly emerging drug resistant strains against the new class of anti-malarial drugs are major threat to control the disease burden worldwide. Helicases are present in every organism and have important role in various nucleic acid metabolic processes. Previously we have reported the presence of three parasite specific helicases (PSH) in *Plasmodium falciparum* 3D7 strain. Here we present the detailed biochemical characterization of PfPSH2. PfPSH2 is DNA and RNA stimulated ATPase and is able to unwind partially duplex DNA and RNA substrates. It can translocate in both 3′ to 5′ and 5′ to 3′ directions. PfPSH2 is expressed in all the stages of intraerythrocytic development and it is localized in cytoplasm in *P*. *falciparum* 3D7 strain. The dsRNA mediated inhibition study suggests that PfPSH2 is important for the growth and survival of the parasite. This study presents the detailed characterization of PfPSH2 and lays the foundation for future development of PfPSH2 as drug target.

## Introduction

Malaria is one of the most widespread and deadly parasitic disease caused by the *Plasmodium* parasite and transmitted to the humans by the bite of female *Anopheles* mosquito^1^. There are five main malaria causing species of *Plasmodium* such as *Plasmodium falciparum*, *Plasmodium vivax*, *Plasmodium ovale*, *Plasmodium malariae* and *Plasmodium knowlesi*^2^. *P*. *falciparum* is the major threat to the mankind because it causes most severe form of the disease^2,3^. The impact of the threat to humanity due to malaria can be analyzed by WHO report, which reveals millions of new cases every year^4^. Due to the efforts made by WHO, the number of cases of malaria have been reduced but it still remains a challenge because of the emergence of multiple drug-resistant parasites^5^. The artemisinin-based combination therapy (ACT) is used currently due to the loss of effectiveness of the old conventional therapeutics regime, which included the use of combination of sulfadoxine-pyrimethamine (SP) and chloroquine^6^. ACT has been successfully used since past two decades and it has shown great progress in malaria control throughout the world. The reports of declining rate of sensitivities of the parasite to ACT poses a major setback for the malaria control efforts. The first report of resistance of parasite to ACT was from Western Combodia^7–9^ but now ACT failures have been reported in several regions of Asia such as Thailand, Myanmar, Vietnam and China^5,10–13^. There is an urgent need to identify new drug targets to curtail the parasite growth and reduce malaria burden worldwide.

Helicases separate double helix of the DNA strands or secondary structures of RNA by utilizing energy harnessed from ATP hydrolysis and therefore all the helicases contain intrinsic nucleic-acid-dependent ATPase activity^14^. The importance of helicases is further strengthened because they are encoded by a major fraction of the prokaryotic and eukaryotic genome^15,16^. On the basis of conserved amino acid signature motifs, the helicases have been divided into six superfamilies’ SF1-SF6^17^. The most studied superfamilies of helicases are SF1 and SF2, which also show similarities in the conserved amino acid signature motifs^18,19^. The conserved signature motifs contribute to form the catalytic core that folds into two Rec-A like domains responsible for ATPase and helicase activity^20^. SF2 is the largest of all other superfamily’s and contains DExD/H box proteins, which have been named on the basis of amino acids present in motif II. The DExD/H box proteins have a striking similarity in their conserved signature motifs^17,21^. Despite having similarities in the core domains, most of the helicases contain variable flanking N and C-terminal extensions, which provide some additional domains for other functions^22–24^. The N and C-terminal extensions help in recruitment of the other interacting protein partners to the complex responsible for the specific function inside the cell^25^. Helicases regulate major biological pathways such as genome replication, translation, repair, mRNA splicing and transcription in all the organisms including malaria parasite^26,27^. Furthermore, helicases are also involved in cross-talk with various other biological pathways such as autophagy, apoptosis and homeostasis regulations^28–30^. Helicases have been reported as potential drug target because their down regulation results in curtailing parasite growth due to inhibition of the major biological pathways of the parasite^31^. The studies done on yeast show that helicases are essential enzymes and the loss of one helicase cannot be replaced by over-expression of the other helicase^32,33^.

The genome-wide analysis of *P*. *falciparum* revealed that it contains novel helicases, which are specific to the parasite and their homologs are not detectable in the human host^34^. Previously we have reported the biochemical characterization of PfUvrD, which is specific to the parasite, and is absent from the human host due to its prokaryotic nature. PfUvrD is an important component of mismatch repair complex of *P*. *falciparum*^35^. PfUvrD is a DNA helicase and unwinds DNA duplex in 3′ to 5′ direction and it colocalizes and interacts with PfMLH, which modulates its unwinding activity. UvrD is expressed in schizont stage of the intraerythrocytic developmental stages of the parasite development and it is localized in the nucleus in the parasite^36^. Previously we have also reported the detailed biochemical characterization of parasite specific helicase 3 (PfPSH3), which is only present in the parasite and is absent from the human host. PfPSH3 is an ATPase and unwinds duplex DNA in 3′ to 5′direction. PSH3 is a nucleocytoplasmic protein which is present in the nucleus in early intraerythrocytic developmental stages of parasite and in later stages it is present in the cytoplasm. The colocalization studies with cytoplasmic RNA helicase PfDOZI confirmed its presence in the cytoplasm^37^.

In this manuscript, we report the detailed biochemical characterization of purified recombinant parasite specific helicase 2 (PfPSH2) (PF3D7_1202000) from *P*. *falciparum* 3D7 strain. The ~105 kDa protein encoded by the full-length gene (2634 base pair) was used for all the biochemical assays. The biochemical characterization shows that PfPSH2 exhibits DNA and RNA dependent-ATPase activity. PfPSH2 also shows dual helicase activity because it can unwind partially duplex DNA and RNA substrates. Furthermore, PfPHS2 is a bi-directional helicase since it can unwind the DNA duplex in both the 5′-3′ and 3′-5′ directions. PfPSH2 mutant (PfPSH2M; K209E) with substitution in motif I (GTGKT) at conserved lysine to glutamic acid residue was generated. Purified PfPSH2M lacks significant ATPase and helicase activity. The immunofluorescence assay of PfPSH2 indicates that it is partially localized in the nucleus in trophozoite stage only. In other intraerythrocytic developmental stages it is mainly localized in the cytoplasm and it colocalizes with PfPABP (Pf poly A binding protein) in the cytoplasm. The results of dsRNA mediated growth inhibition assay revealed the morphological and growth defect changes when the parasite was treated with PfPSH2 specific dsRNA. The results of semi quantitative real time PCR suggest that the observed growth defect of the parasite was most likely due to the reduction of PfPSH2 specific mRNA level after application of PfPSH2 specific dsRNA.

## Results

### In silico analysis and structural modeling of PfPSH2

**Figure 1:**
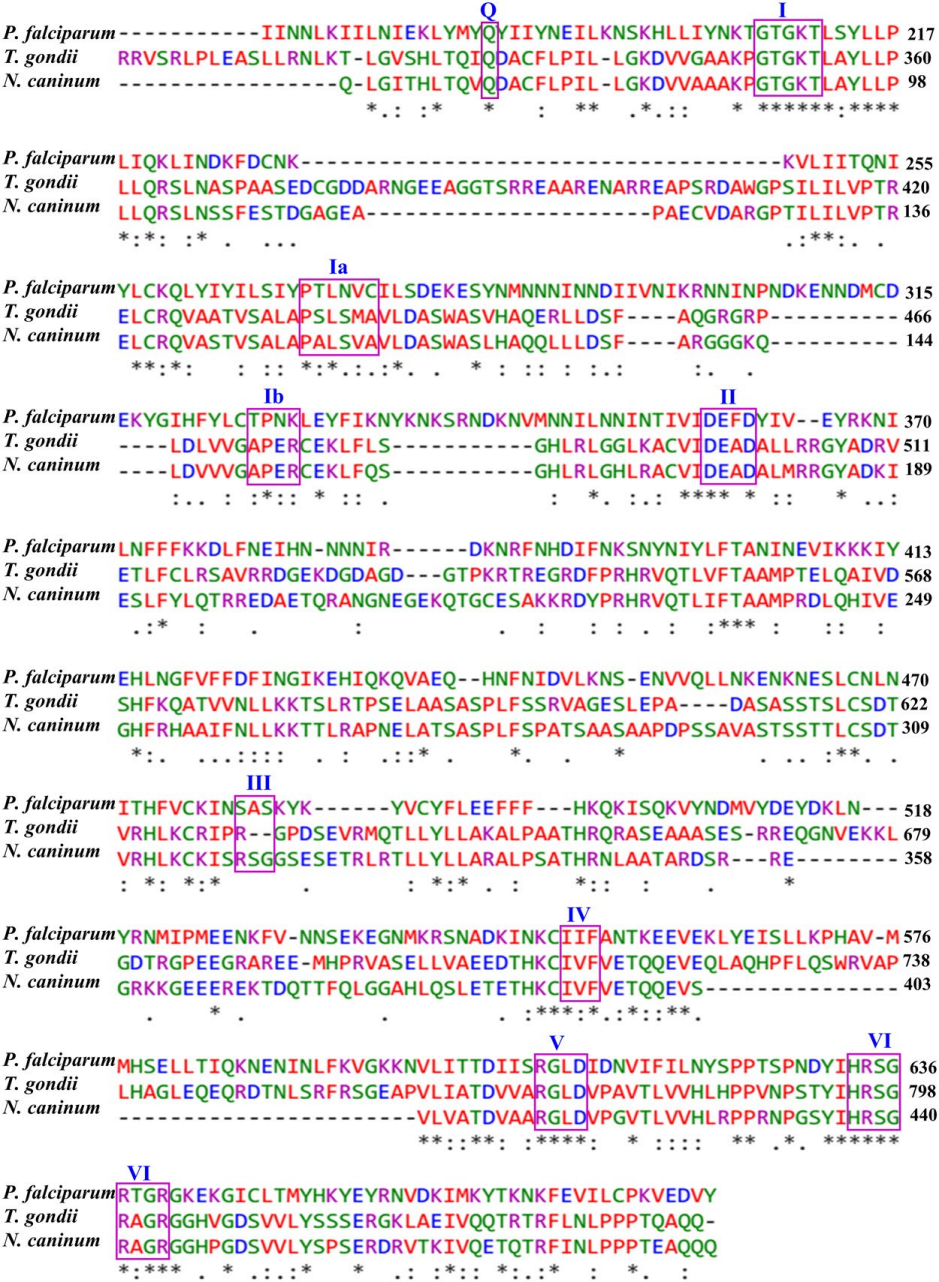
Multiple Sequence Alignment of the amino acid sequence of the PfPSH2 with orthologs present in apicomplexans *Toxoplasma gondii* and *Neospora caninum*. The alignment was done using clustal omega program (www.ebi.ac.uk/Tools/msa/clustalo/). For motif representation, all signature motifs of PfPSH2 are highlighted and boxed in purple color and the name of each motif (from I to VI) is written in roman numerals.

OrthoMCL (www.orthomcl.org)^38^ database analysis revealed that PfPSH2 belongs to orthologous group OG5_153588, which contains a total of nine orthologs including PfPSH2. The size of PfPSH2 is ~105 kDa and its core region is from 185 to 637 amino acids and it contains ~184 amino acids N terminal and ~243 amino acids C terminal extension. Apart from *Plasmodium* species, proteins from only two other apicomplexans show little homology to PfPSH2 i.e. *T*. *gondii* (~21%) and *N*. *caninum* (~22%). Using the usual bioinformatics tools, we were unable to detect a protein homologous to PfPSH2 in other eukaryotes. PfPSH2 contains all the nine conserved signature motifs and was used for multiple sequence alignment with orthologs present in other organisms using Clustal Omega^39^ (http://prosite.expasy.org/prosite.html) (Fig. 1, and Supplementary Fig. 1A,B). PfPSH2 shows variation in conserved signature motifs from its orthologs. In conserved motif II, PfPSH2 contains F (phenylalanine) whereas usually A (alanine) is present (DEAD) in this motif (Fig. 1).

**Figure 2:**
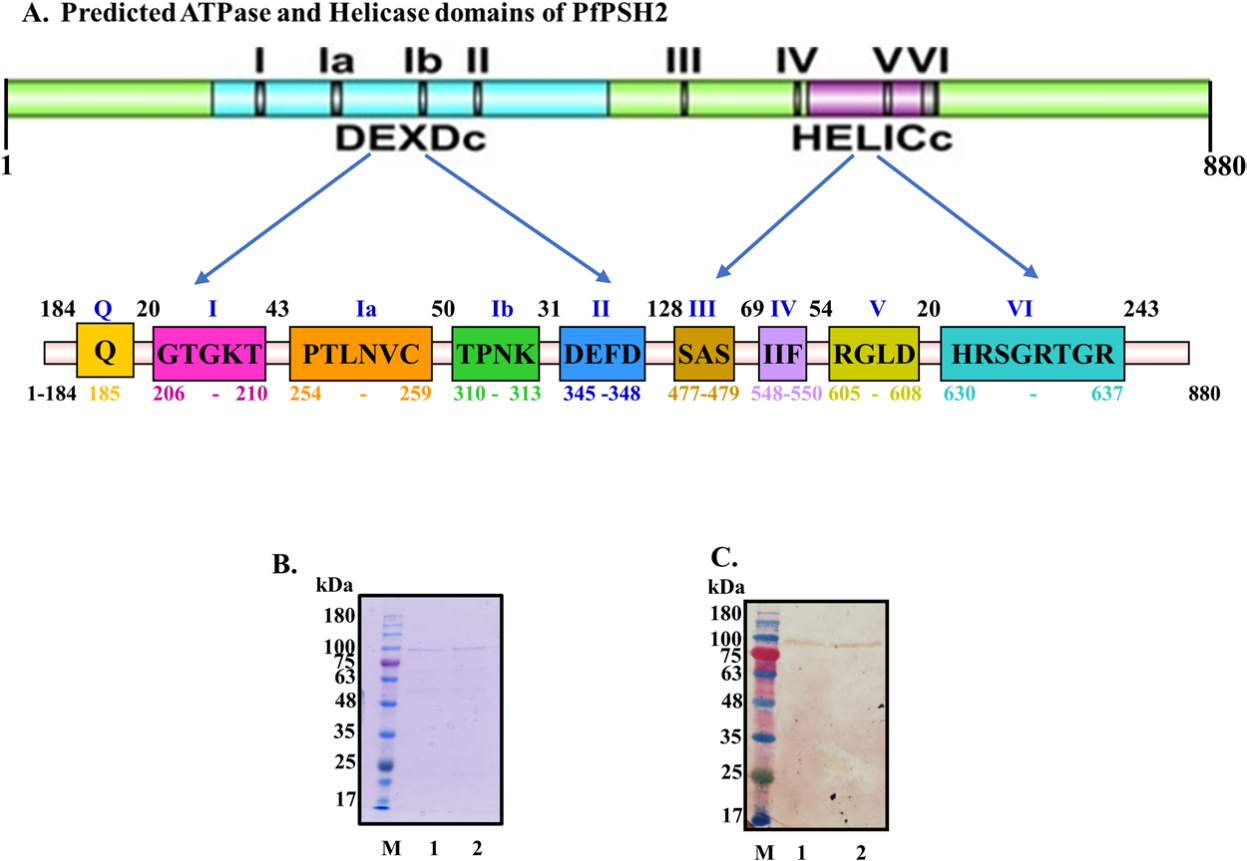
(**A**) Schematic representation of signature motifs of DEAD box family of PfPSH2. All the domains are presented with their respective amino acid positions; (**B**) SDS PAGE analysis. Commassie blue stained gel. Lane M is molecular weight marker and lanes 1 and 2 are purified PfPSH2 and PfPSH2M proteins (~105 kDa); (**C**) Western Blot analysis. Lane numbers are similar to B.

The Prosite (www.http://prosite.expasy.org/prosite.html)^40^ software was used to predict domain organization of PfPSH2 and position of both ATPase domain (from amino acid 206–479) and helicase C terminal domain (from amino acid 548–637) (Fig. 2A). All the nine conserved signature motifs of DEAD box helicase such as Q, I, Ia, Ib, II, III, IV, V, and VI are present within both ATP binding and helicase C-terminal domains of PfPSH2 (Fig. 2A). To determine the structural similarity of the PfPSH2 with the structures available in PDB (Protein data bank) and to predict the structure of PfPSH2 the Swissmodel homology modelling server (http://swissmodel.expasy.org/) was used^41,42^. The template was selected on the basis of maximum coverage and identity with the PfPSH2 amino acid sequence and the template selected was 5ivl.1.A (Cold shock helicase A (CshA)). CshA is an ATP dependent DEAD box helicase involved in mRNA turnover and its dimeric form helps ribonuclease for RNA decay^43^. Pdb files of the template and predicted protein model were retrieved and were converted to images and presented with the help of UCSF Chimera software (Supplementary Fig. 1C, panels i-iii)^44^.

### Purification of PfPSH2N, PfPSH2, and Mutant PfPSH2 (PfPSH2M) proteins

PfPSH2 is 2643 nucleotides long and it encodes for 880 amino acids long protein of ~105 kDa. To generate polyclonal antibodies, the smaller N-terminal fragment; PfPSH2N was cloned. PfPSH2N is 537 nucleotides long and it encodes for 179 amino acids long protein of ~28 kDa. Both PfPSH2N and PfPSH2 were amplified (Supplementary Fig. 2A) using gene-specific primers described in Supplementary Table 1. The mutant of full-length PfPSH2 (PfPSH2M) was generated and mutation of desired nucleotide was confirmed by DNA sequencing. In motif I (GTGKT) of ATPase domain, the conserved lysine was mutated to glutamic acid (GTGET) to generate the mutant (PfPSH2M; K209E). All the proteins were purified utilizing the Ni-NTA affinity chromatography. The purified fractions of PfPSH2, PfPSH2M and PfPSH2N were then separated on SDS PAGE (Fig. 2B, lanes 1–2 and Supplementary Fig. 2B,D) and subjected to western blot analysis using anti-6X his antibody. The western blot analysis clearly indicated the presence of PfPSH2, PfPSH2M and PfPSH2N in the purified fractions (Fig. 2C, lanes 1–2 and Supplementary Fig. 2C, lane 1 and 2E).

### ATPase activity assay of PfPSH2 and its mutant PfPSH2M

**Figure 3:**
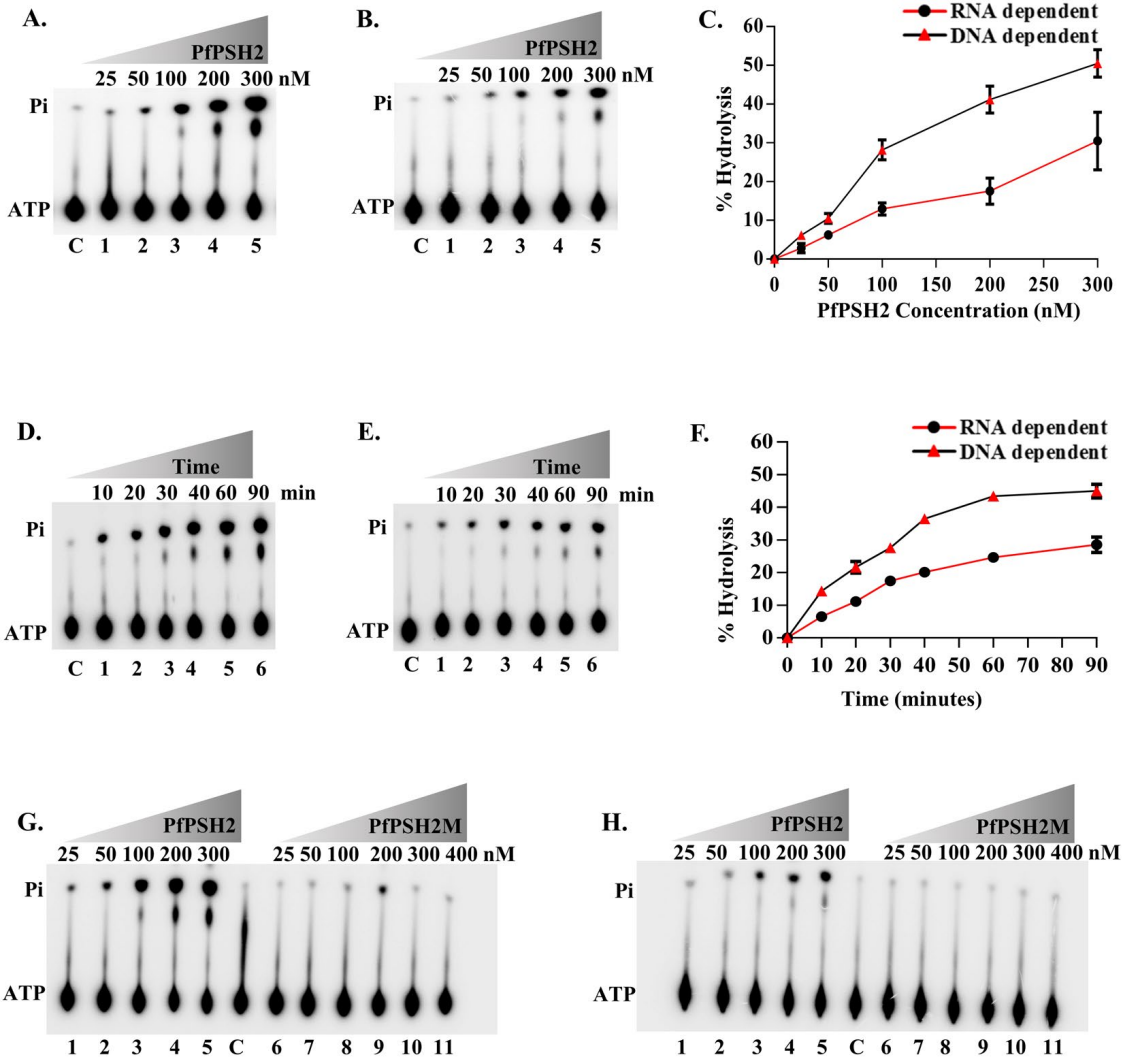
ATPase activity analysis of PfPSH2 and PfPSH2M. (**A**) ATPase activity in the presence of DNA with increasing concentration (25 nM to 300 nM) of PfPSH2 protein (lanes 1–5); the experiment was repeated at least three times; (**B**) ATPase activity in the presence of RNA with increasing concentration (25 nM to 300 nM) of PfPSH2 protein (lanes 1–5), the experiment was repeated at least three times; (**C**) Graphical representation of quantitative data of Fig. 3A,B, the triangle and circle data points depict % ATP hydrolysis in the presence of DNA or RNA, respectively. (**D**) Time dependent ATPase activity of PfPSH2 (lanes 1–6) in the presence of DNA; the experiment was repeated at least three times (**E**) Time dependent ATPase activity of PfPSH2 (lanes 1–6) in the presence of RNA; experiment was repeated at least three times; (**F**) Graphical representation of quantitative data of Fig. 3D,E, data points with triangle and circle depict % ATP hydrolysis in the presence of DNA or RNA at different time intervals; (**G**) ATPase assay with increasing protein concentration (25 nM to 400 nM) of PfPSH2 and PfPSH2M in the presence of DNA (**H**) ATPase assay with increasing protein concentration (25 nM to 400 nM) of PfPSH2 and PfPSH2M in the presence of RNA. Lane C in panels A, B, D, E, G and H represents control reaction without protein.

For the analysis of ATPase activity, radio labelled (γ^32^P) ATP was used and the release of γ^32^P labeled phosphate (Pi) was measured by using varying concentration of purified PfPSH2 (25 nM to 300 nM) with DNA or RNA as a cofactor (Fig. 3A,B). The results indicate that PfPSH2 shows concentration-dependent ATPase activity in the presence of DNA with a linear increase in activity. ATP hydrolysis increased with increasing concentration of protein and at 300 nM PfPSH2 the hydrolysis is ~50% (Fig. 3A, lanes 1–5 and Fig. 3C). With RNA as cofactor, PfPSH2 also showed concentration-dependent ATPase activity and the hydrolysis reached to ~30% at 300 nM (Fig. 3B, lanes 1–5 and Fig. 3C). It is interesting to note that with DNA as a cofactor, the ATPase activity increased linearly with increase in protein concentration of PfPSH2 (Fig. 3A, lanes 1–5 and Fig. 3C) but with RNA as a cofactor the activity did not increase beyond ~35% ATP hydrolysis at higher protein concentration (Fig. 3B, lanes 1–5 and Fig. 3C). At lower protein concentrations the ATPase activity of PfPSH2 in the presence of DNA or RNA is similar but the difference was observed when the concentration was increased from 100 nM to 300 nM. The time-dependent ATPase activity was also measured with varying incubation time of reaction using fixed protein concentration of 100 nM in the presence of DNA or RNA. The results indicate that PfPSH2 shows time-dependent ATPase activity in the presence of DNA or RNA. In the presence of DNA, PfPSH2 exhibits ATPase activity within 10 minutes with ~14% ATP hydrolysis and a maximum of ~44% ATP hydrolysis was observed after 90 minutes (Fig. 3D, lanes 1–6 and Fig. 3F). In the presence of RNA, PfPSH2 exhibits a maximum of ~28% ATP hydrolysis after 90 minutes (Fig. 3E, lanes 1–6 and Fig. 3F). The ATPase activity of the mutant PfPSH2 (PfPSH2M) was analyzed using a similar procedure. The results indicate that it exhibits no detectable ATPase activity in the presence of either DNA or RNA respectively, as compared to wild-type PfPSH2 using identical concentrations of protein (Fig. 3G, lanes 1–5 and lanes 6–11 and Fig. 3H lanes 1–5 and lanes 6–11, respectively).

### DNA helicase activity assay of PfPSH2 and its mutant PfPSH2M

**Figure 4:**
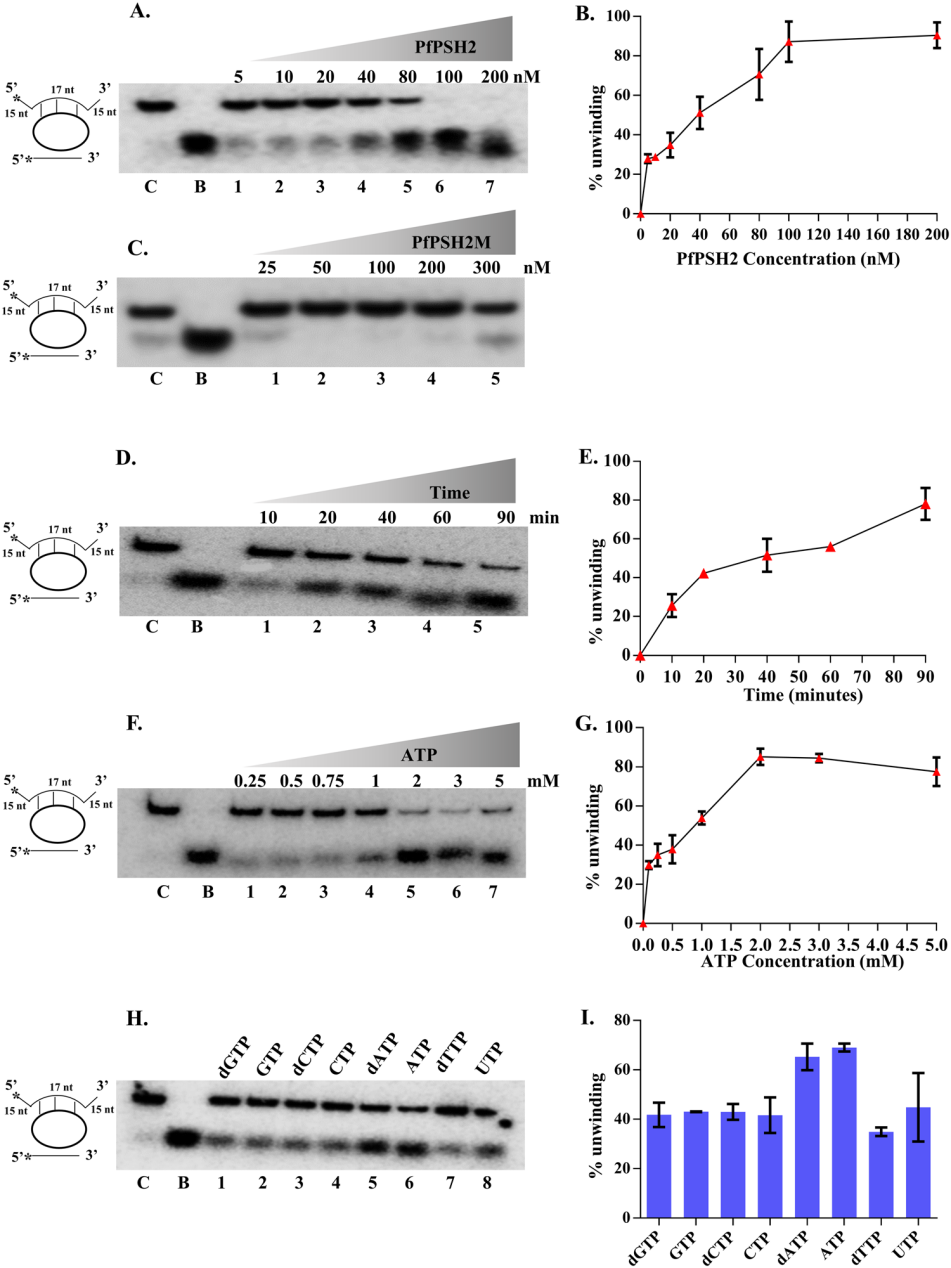
DNA Helicase activity assay of PfPSH2. (**A**) Lanes 1–7, reactions with increasing concentration (5 nM to 200 nM) of PfPSH2 protein; the experiment was repeated at least three times; (**B**) Graphical representation of quantitative data of Fig. 4A. (**C**) Lanes 1–5, reactions with increasing concentration (25 nM to 300 nM) of PfPSH2M protein; the experiment was repeated at least two times; (**D**) Lanes 1–5, show time dependent helicase activity with fixed concentration (80 nM) of PfPSH2 protein; the experiment was repeated at least two times; (**E**) Graphical representation of quantitative data of Fig. 4D. (**F**) Lanes 1–7, helicase activity of PfPSH2 at different concentrations of ATP (0.25 M to 5 M); the experiment was repeated at least two times. (**G**) Graphical representation of quantitative data of Fig. 4F. (**H**) Helicase activity of PfPSH2 in the presence of different nucleotides/deoxynucleotides triphosphates such as dGTP, GTP, dCTP, CTP, dATP, ATP, dTTP and UTP (lanes 1–8); the experiment was repeated at least two times; (**I**) Graphical representation of quantitative data of Fig. 4H. In A, C, D, F and H, lane C is control reaction without protein and lane B is boiled substrate.

In order to assess the unwinding ability of purified PfPSH2 partially duplex DNA and RNA substrates were used. The partially duplex DNA substrate was prepared using the method described in Supplementary Fig. 3. To determine the concentration-dependent unwinding of partially duplex DNA substrate, various concentrations of PfPSH2 ranging from 5 nM to 200 nM were used and the results show that maximum of ~90% unwinding was observed at 200 nM (Fig. 4A, lanes 1–7 and Fig. 4B). The unwinding of partially duplex substrate was ~25% with as low as 5 nM PfPSH2 and it did not significantly increase initially with increase in protein concentration up to 20 nM (Fig. 4A, lanes 1–3 and Fig. 4B). It is interesting to note that the unwinding increased logarithmically with increasing concentration of protein from 20 nM to 80 nM and a maximum of ~89% unwinding was observed with 100 nM protein concentration (Fig. 4A, lanes 4–7 and Fig. 4B). Varying concentrations from 25 nM to 300 nM of PfPSH2M were also used for determining the unwinding of partially duplex DNA substrate. There was no significant unwinding of the partially duplex DNA substrate even at highest concentration of PfPSH2M (Fig. 4C, lanes 1–5). To analyze the time-dependent DNA helicase activity of PfPSH2, fixed concentration (80 nM) of protein was used and the reaction was incubated at different time points. The results indicate that within 20 minutes, PfPSH2 starts unwinding the partially duplex substrate and maximum unwinding of ~78% was achieved in 90 minutes (Fig. 4D, lanes 1–5 and Fig. 4E).

### ATP concentration-dependent and Nucleotide dependent assays for DNA helicase activity

To analyze the optimal concentration of ATP required by PfPSH2 to unwind partially duplex DNA substrate; varying concentrations of ATP (0.25 mM to 5 mM) were used in reaction mixture using fixed concentration (80 nM) of PfPSH2. The results indicate that maximum of ~77% unwinding was achieved at 2 mM ATP concentration with saturation at 3 mM (Fig. 4F, lanes 1–7 and Fig. 4G). It was also observed that beyond 3 mM ATP concentration the unwinding activity of PfPSH2 started decreasing (Fig. 4F, lanes 6–7 and Fig. 4G). The results suggest that the optimal range of ATP concentration for PfPSH2 unwinding is 1–3 mM. Further, the nucleoside tri phosphates (NTPs) dependence of PfPSH2 was analyzed using all the NTPs and dNTPs (deoxy nucleoside tri phosphates). Different NTPs/dNTPs such as CTP, GTP, UTP, dCTP, dGTP and dTTP were used in place of ATP in the reaction mixture. The results show that PfPSH2 prefers ATP and dATP for unwinding the duplex substrate with ~69% and ~65% unwinding, respectively (Fig. 4H, lanes 5–6 and Fig. 4I). In the presence of other NTPs/dNTPs, PfPSH2 showed lesser unwinding activity in the range of ~30–40% (Fig. 4H, lanes 1–4, and lanes 7–8 and Fig. 4I).

### Direction specificity of PfPSH2

**Figure 5:**
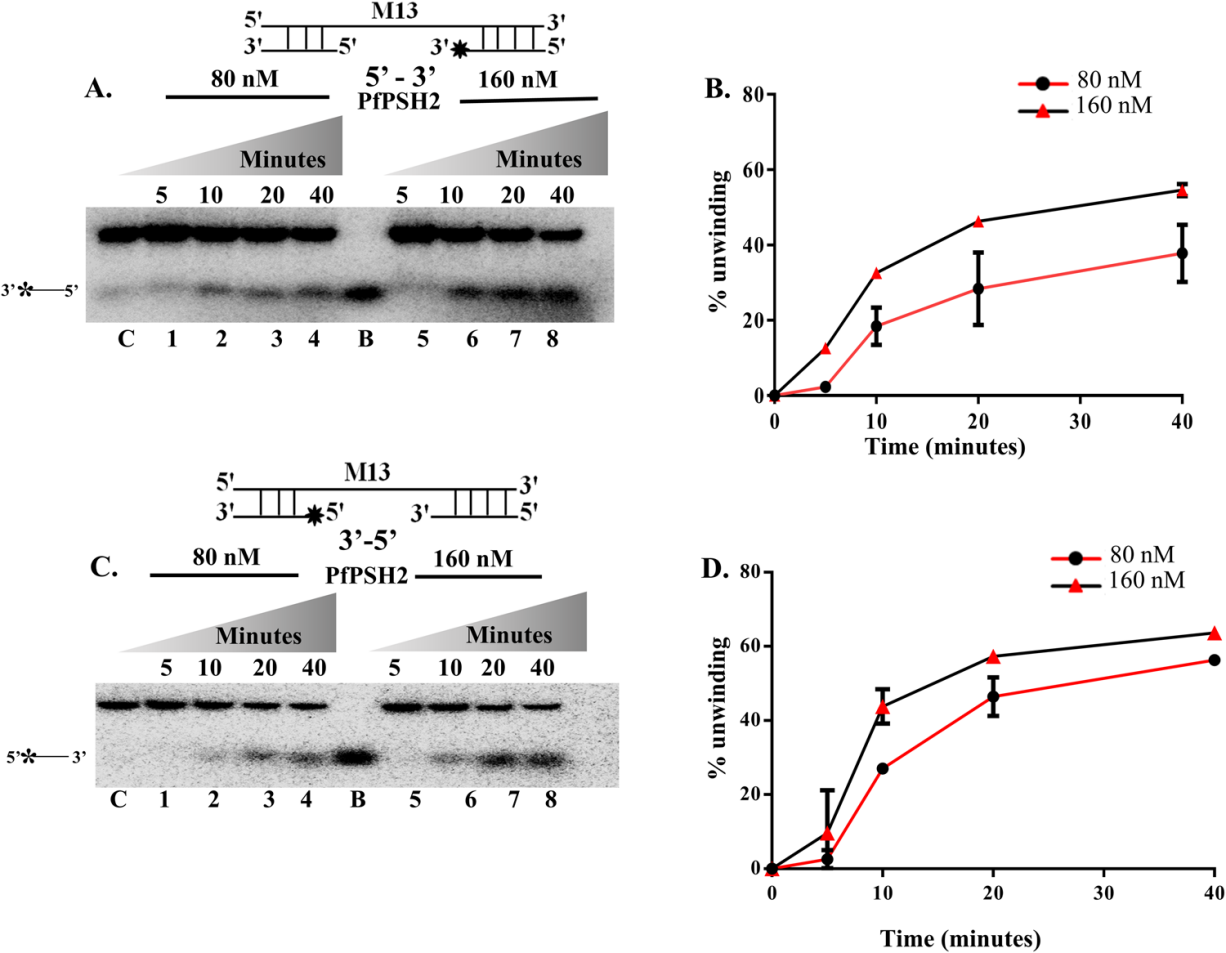
Direction specific helicase activity assay. (**A**) Helicase activity of PfPSH2 with the 5′ to 3′ direction- specific substrate. Lanes 1–4 are reactions with 80 nM of PfPSH2 and lanes 5–8 are reactions with 160 nM of PfPSH2, respectively at various time points. The experiment was repeated at least two times; (**B**) Graphical representation of quantitative data of Fig. 5A, data points with circles and triangles depict % unwinding with 80 nM and 160 nM of PfPSH2, respectively. (**C**) Helicase activity of PfPSH2 with the 3′ to 5′ direction specific substrate. Lanes 1–4 are reactions with 80 nM of PfPSH2 and lanes 5–8 are reactions with 160 nM of PfPSH2, respectively at various time points. The experiment was repeated at least two times; (**D**) Graphical representation of quantitative data of Fig. 5C, data points with circles and triangles depict % unwinding of 3′-5′ direction substrate with 80 nM and 160 nM of PfPSH2, respectively. In A and C, lane C is control reaction without protein and lane B is boiled substrate.

The directionality of the movement of helicase on the substrate is determined by its loading and movement on the substrate towards 5′ or 3′ end^45^. To determine direction specificity of PfPSH2, two different direction-specific substrates were prepared and used (Supplementary Fig. 3). The unwinding activity was analyzed using two different concentrations of PfPSH2, 80 nM and 160 nM, at different time points. The results show that PfPSH2 unwound both the substrates, and with 5′ to 3′ direction-specific substrate maximum unwinding observed was ~54% in 40 minutes (Fig. 5A, lanes 1–4 and lanes 5–8 and Fig. 5B). With 3′ to 5′ direction-specific substrate, the unwinding observed using PfPSH2 was around ~63% in 40 minutes (Fig. 5C, lanes 1–4 and lanes 5–8 and Fig. 5D). These results suggest that PfPSH2 exhibits concentration and time-dependent bipolar DNA unwinding activity.

### RNA helicase activity assay of PfPSH2 and its mutant PfPSH2M

**Figure 6:**
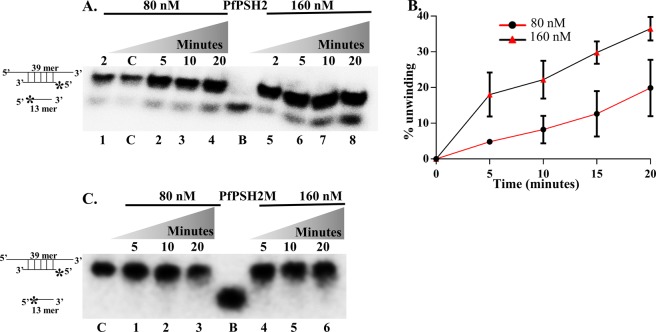
RNA helicase activity assay. (**A**) RNA helicase activity of PfPSH2. Lanes 1–4 are reactions with 80 nM and lanes 5–8 are reactions with 160 nM of PfPSH2, respectively at various time points. The experiment was repeated at least two times; (**B**) Graphical representation of quantitative data of Fig. 6A, data points with circles and triangles depict % unwinding of RNA substrate with 80 nM and 160 nM of PfPSH2, respectively. (**C**) RNA helicase activity of PfPSH2M. Lanes 1–4 are reactions with 80 nM and lanes 5–8 are reactions with 160 nM of PfPSH2M, respectively at various time points. The experiment was repeated at least two times. In A and C, lane C represents control reaction without protein and lane B is boiled substrate.

The RNA helicase activity of PfPSH2 was determined using a partially duplex RNA substrate that was prepared as described in methods section. To determine the concentration and time-dependent unwinding of duplex RNA substrate by PfPSH2, the assay was performed using two different protein concentrations (80 nM and 160 nM) at different time intervals (5 to 20 minutes). The results indicate that PfPSH2 shows time and concentration-dependent unwinding of partially duplex RNA substrate with a minimum of ~4.5% and a maximum of ~36% unwinding, respectively (Fig. 6A, lanes 1–8 and Fig. 6B). The RNA helicase activity of PfPSH2M was also checked with the same substrate and the results indicate that the mutant protein contains no detectable RNA unwinding activity (Fig. 6C, lanes 1–6).

### Localization and *in vivo* expression of PSH2

**Figure 7:**
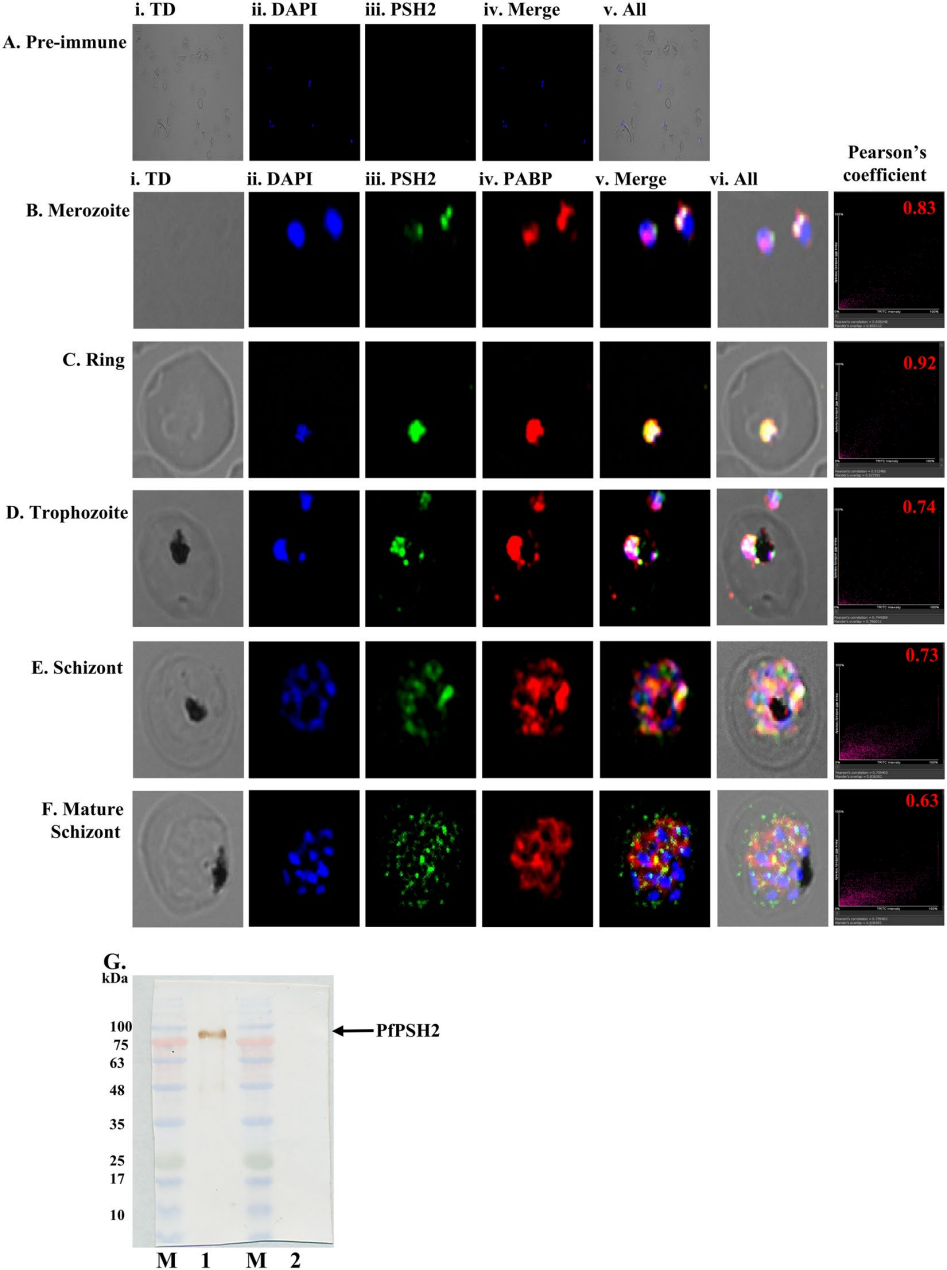
Localization of PSH2 in different intra-erythrocytic stages of *P*. *falciparum*. The cells were fixed and stained with pre-immune sera or anti-PfPSH2 antisera and anti-PfPABP antisera followed by Alexa fluor 488 and Alexa fluor 594-conjugated secondary antibodies and then counterstained with DAPI. In each panel, single confocal image of each stage is shown. (**A**) Staining with pre-immune sera (i) phase contrast (TD) image; (ii) image of cell stained with DAPI (blue); (iii) pre-immune sera PSH2; (iv) PSH2 + DAPI (v) All merged; (**B–H**) Staining with anti-PSH2 and anti-PABP sera (**B**) merozoite stage, (**C**) ring stage, (**D**) trophozoite stage, (**E**) schizont stage, (**F**) schizont stage (i) phase contrast (TD) image; (ii) image of cell stained with DAPI (blue); (iii) immunofluorescent stained cell (PSH2); (iv) immunofluorescent stained cell (PABP); (v) Merged image of panel ii, iii and iv; (vi) Merged image of panel i-v (vii) Pearson’s correlation coefficients of merged images are written. (**G**) Western blot analysis using lysate of mixed stage *P*. *falciparum* 3D7 strain culture. Lane M is prestained protein molecular weight marker and lane 1 is parasite lysate immunoprecipitated with anti-PfPSH2N antiserum and lane 2 is parasite lysate immunoprecipitated with pre-immune serum.

The localization of PfPSH2 in the parasite was checked at various intraerythrocytic asexual developmental stages of *P*. *falciparum* 3D7 strain. To study the localization immunofluorescence assay (IFA) was done using antibodies generated against PfPSH2N. DAPI was used to locate nucleus and Alexa Fluor 488 dye conjugated secondary antibody was used to localize PSH2. Pre-immune sera were also used with Alexa Fluor 488 dye-conjugated secondary antibody and the results show that pre-immune sera did not stain the parasite because no Alexa flour 488 signal was detected and only DAPI staining was visible (Fig. 7A, panels i-v). The confocal images obtained using the anti-PfPSH2N sera suggest that the localization of PfPSH2 is mostly in the cytoplasm and it is very less in the nucleus at some intraerythrocytic developmental stages of the parasite (Supplementary Fig. 4A–H, panels i-v). In order to determine the specific localization of PfPSH2 in the cytoplasm, the colocalization was done using the antibodies specific to the cytoplasmic protein PfPABP (poly(A)-binding protein)^46^. The results revealed that PfPSH2 and PfPABP colocalize in merozoite stages of the parasite and Pearson’s coefficient is 0.83 (Fig. 7B, panels i-vi). Similarly, in ring stage the colocalization is even more visible and Pearson’s coefficient is 0.92 (Fig. 7C, panels i-vi). In case of trophozoite stage some nuclear localization of PfPSH2 is visible and in this stage of intraerythrocytic development, PfPSH2 also colocalizes with PfPABP with the Pearson’s coefficient value of 0.74, which is less than in merozoite and ring stages (Fig. 7D, panels i-vi). In schizont stages PfPSH2 shows punctate type localization that is scattered in the cytoplasm and it also colocalizes with PfPABP with the Pearson’s coefficient value of 0.73 and 0.63, respectively (Fig. 7E and F, panels i-vi).

The mixed parasite lysate and the preimmune serum and anti-PfPSH2N sera were used to check the level of expression of PSH2 in the *P*. *falciparum* 3D7 parasite lysate using the protocol described (Supplementary Fig. 5). There was no detectable protein in the precipitate of the preimmune serum (Fig. 7, lane 2). In the precipitate of PfPSH2N antibodies, a single band was detectable (Fig. 7, lane 1).

### dsRNA mediated growth inhibition and mRNA expression analysis of dsRNA treated cultures

**Figure 8:**
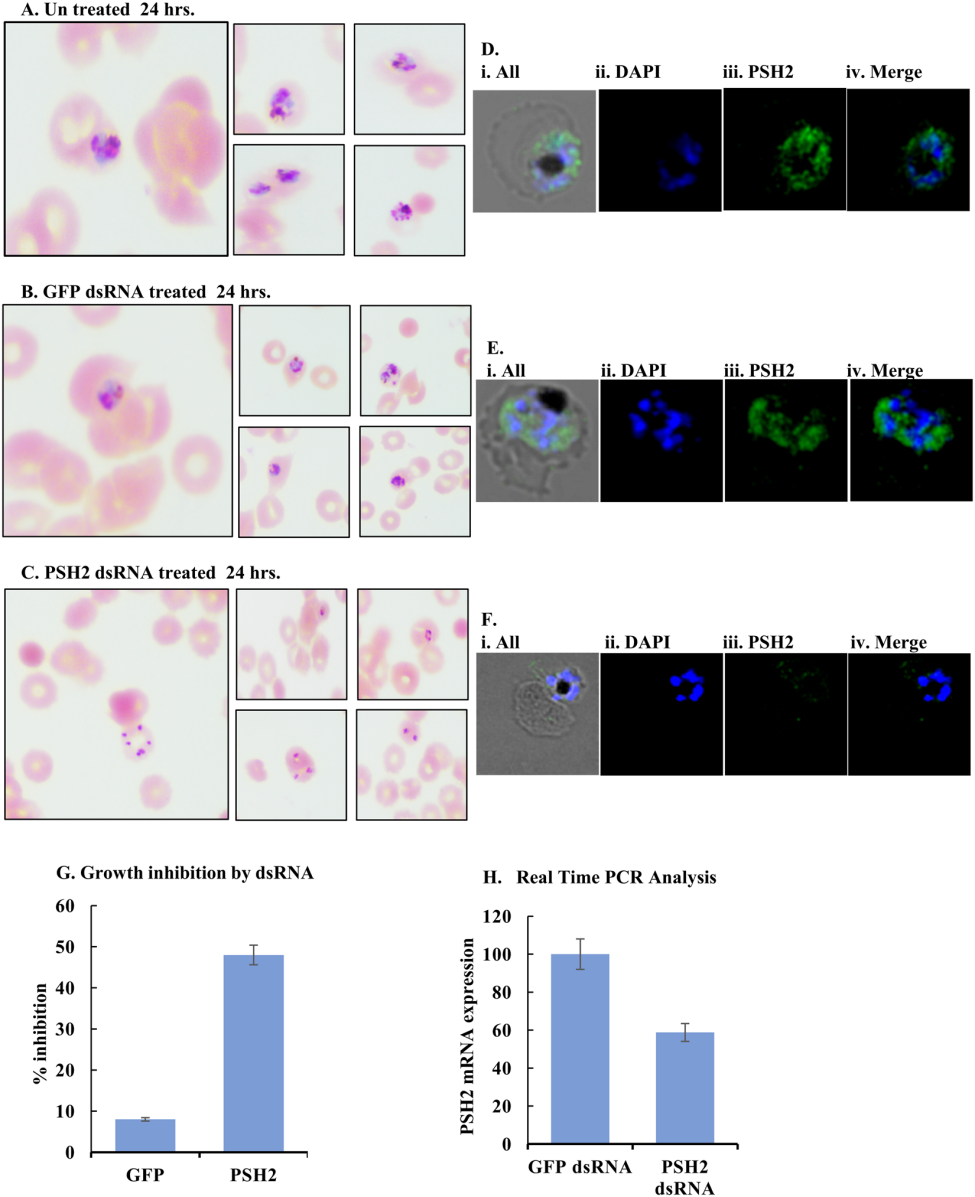
Effect of dsRNA on parasite growth. (**A–C**) Giemsa-stained parasite infected RBCs at 24 hours (**A**) before dsRNA treatment (Control); (**B**) After GFP-dsRNA treatment; (**C**) After PfPSH2 dsRNA treatment; (**D–F**) Immunofluorescence microscopy with PfPSH2 anti-sera; (**D**) untreated parasite (**E**) GFP dsRNA treated parasite (**F**) PfPSH2 dsRNA treated parasite; In D-F (i) TD and merge image; (ii) image of cell stained with DAPI (blue); (iii) immunofluorescent stained cell (PSH2); (iv) image of cell stained with DAPI + PSH2; (**G**) Growth inhibition of parasite calculated by manual microscopic counting of Giemsa stained slides (**H**) Real-time PCR analysis results of GFP dsRNA treated samples and PfPSH2 dsRNA treated samples. The experiments were repeated at least three times.

To analyze the effect of PfPSH2 dsRNA on *P*. *falciparum* 3D7 growth, sorbitol synchronized parasite culture at ~1% parasitemia and 2% hematocrit were used. The PfPSH2 and green fluorescent protein (GFP) dsRNAs were prepared using the method described in Supplementary Fig. 6A,B. The synchronized parasite culture was then treated with 20 μg/ml of PfPSH2 dsRNA and unrelated GFP dsRNA was used as control. Both of these dsRNA treated cultures were then observed for 48 hours. The smears were prepared every 24 hours and used for Giemsa staining and counting parasitemia. The results suggest that the untreated culture grew normally (Fig. 8A). The GFP dsRNA treated parasite culture also had no significant growth inhibition after 24 hours of dsRNA treatment (Fig. 8B). The microscopic counting of these stained smears revealed abnormality in the morphology of parasite and inhibition of parasite growth after 24 hours in parasite culture treated with PfPSH2 dsRNA (Fig. 8C). The immunofluorescence microscopy was also done to visualize PfPSH2 inside the parasite after dsRNA treatment by using anti-PfPSH2N sera. The results show that PfPSH2 is insignificantly visible in PSH2 dsRNA treated parasite in comparison to the untreated and GFP dsRNA treated parasites (Fig. 8D–F panels i-iv). After 48 hours, PfPSH2 dsRNA treated cultures showed more than ~48% growth inhibition and severe morphological abnormalities such as shrinkage of nucleus and cytoplasm of parasite, whereas GFP dsRNA treated culture showed only ~8% growth inhibition (Fig. 8G).

In order to analyze the effect of dsRNA on the transcript level of targeted gene, a parallel experiment was also performed. The unsynchronized parasite culture was treated with 20 μg/ml of PfPSH2 dsRNA and with unrelated dsRNA of GFP. Total RNA was isolated from the dsRNA treated parasite cultures using RNeasy mini RNA isolation kit. Equal amount of RNA (1 μg) was used from each sample to synthesize cDNAs. These synthesized cDNAs were used for real time PCR experiment using primers described in Supplementary Table 1 and SYBR Green qPCR master mix as described in methods section. Pf18s rRNA gene was also amplified and used as reference. The results revealed that there is no significant decrease in PfPSH2 mRNA level in GFP dsRNA treated cultures. But the mRNA level of PfPSH2 is significantly decreased in PfPSH2 dsRNA treated cultures when compared with the difference in delta Ct values with 18s rRNA gene and PfPSH2 gene. The results suggest that the mRNA level of PfPSH2 is decreased in PfPSH2 dsRNA treated cultures (Fig. 8H). These observations suggest that the PfPSH2 dsRNA specifically downregulates the mRNA levels of PfPSH2.

### In silico protein-protein interaction prediction

To predict the interacting partners of PfPSH2 in *P*. *falciparum* 3D7; online available interaction prediction software StringDB 10.5 (http://string-db.org/) was used (Supplementary Fig. 7A)^47,48^. The predicted interacting partners of PfPSH2 have probable roles in various cellular pathways such as splicing, mRNA regulation, ribosome biogenesis and exosome mediated RNA decay. The predicted partners are splicing factors Prp22 and Prp43; ribosomal maturation factors pescadillo homolog, Dbp9, Dbp10 and protein SDA1 and mRNA regulation factors DHX36 and helicase SK12W. The description of proteins and the pathways in which they are involved with the string interaction score are shown (Supplementary Fig. 7B).

## Discussion

Genome wide analysis of *P*. *falciparum* 3D7 revealed the presence of several parasite specific helicases, which are absent from the human host^34^. Therefore, it is very important to characterize these helicases and establish their role. Previously detailed biochemical characterization of PfUvrD and PfPSH3 has been reported and both these helicases are specific to parasite and are absent from the human host^35,37^. In this manuscript we have reported the detailed biochemical characterization of PfPSH2, a helicase that is specific to *Plasmodium* species. In silico analysis revealed that apart from *Plasmodium* species, this helicase is present in few other apicomplexans such as *T*. *gondii* and *N*. *caninum* with very low similarity due to conserved core signature motifs. Protein blast search with human host genome database (NCBI) did not show any hit or similarity with the host protein. PfPSH2 is a member of DExD family of DEAD box helicase of superfamily 2. It contains all the seven conserved signature motifs including motif II (DEFD) of DEAD box family of helicases which are important for its ATPase and helicase activity. The biochemical charaterization shows that PfPSH2 is DNA and RNA-dependent ATPase. The observations further suggest that PfPSH2 is able to unwind both DNA and RNA duplex substrates and it is a bipolar helicase that can unwind duplex DNA in both 3′-5′ and 5′-3′ directions. The *Escherichia coli* RecBCD helicase involved in double strand break repair is also a bipolar helicase^49^. Recent report suggests that DEAD box protein DDX43 (HAGE) shows dual RNA-DNA helicase activity^50^. In case of *P*. *falciparum* dual and bipolar helicases have been reported previously. PfDH60 is a dual and bipolar helicase^51^ and is the homolgue of p68 protein^52^. PfH45 is also a dual and bipolar helicase and is important for parasite growth^53^. RNA helicases usually show bidirectional activity because RNAs have secondary structures that are important for their regulatory functions. Evolution of such a bidirectional helicase is energy efficient as it contains both direction specificity in single protein and it can also unwind both DNA and RNA.

The site directed mutant of PfPSH2 was generated by amino acid K209E substitution in motif Ia (GTGKT). PfPSH2M showed insignificant ATPase activity. These results suggest that the lysine in conserved motif Ia is important for the ATPase activity and the loss in ATPase activity yielded negligible DNA or RNA helicase activity in case of PfPSH2M. Previous reports on PfU52 helicase have suggested that mutations in motif Ia (G181A and I182A) and motif Ib (R206A) caused the reduction in ATPase activity in presence of DNA and RNA^54^. The DEAD box helicase Dbp9p mutants at conserved arginine residue of RNA binding motif (R414I, R414T and R414K) show impaired ATPase activity and RNA helicase activity^55^. These observations suggest that the conserved lysine of motif Ia is important for the ATP hydrolysis and its mutation can lead to decrease in ATPase and helicase activity of the protein. PfPSH2 is ATP-dependent helicase and does not unwind duplex substrate well in the presence of other NTPs/dNTPs. Similarly, PfPSH3 which is also a parasite specific helicase prefers only ATP over other NTPs to unwind duplex DNA substrate^37^ It has been reported that there are helicases which can also utilize other NTP hydrolysis for unwinding of DNA duplex. PfUvrD is able to use all the four NTPs to a similar extent to unwind partially duplex DNA substrate^36^. Previously it has been shown that PfWrn can utilize GTP hydrolysis in similar manner to ATP hydrolysis to unwind DNA duplex^56^. The utilization of NTP’s for unwinding of DNA or RNA duplex of all the previously characterized helicases from the *P*. *falciparum* 3D7 are summarized (Table 1).

**Table 1:** Description of NTP utilization and substrate preference of biochemically characterized *Plasmodium falciparum* 3D7 helicases.

S. No.	PlasmoDB ID/Name	NTP/dNTP preference (ATP, GTP, CTP and UTP)	Substrate preference (DNA or RNA)
1.	PF3D7_122710 (PfDH60)	ATP	DNA
2.	PF3D7_146870 (PfH45)	ATP	Both
3.	PF3D7_0209800 (PfU52)	ATP	RNA
4.	PF3D7_0630900 (PfH69)	ATP	Both
5.	PF3D7_1459000 (PfD66)	ATP	Both
6.	PF3D7_0514100 (PfUvrD)	All	DNA
7.	PF3D7_0809700 (PfRuvB1)	ATP	DNA
8.	PF3D7_110600 (PfRuvB2)	ATP	DNA
9.	PF3D7_0320800 (PfDozi)	ATP	RNA
10.	PF3D7_0934100 (PfXPD)	ATP and UTP	DNA
11.	PF3D7_0918600 (PfBLM)	ATP, CTP and dGTP	DNA
12.	PF3D7_1429900 (PfWRN)	ATP and GTP	DNA
13.	PF3D7_ 0807100 (PfPSH3)	ATP	DNA

The identification of subcellular localization is very important for any protein to gain insight of its site of action and involvement in the biological pathways. The biochemical characterization suggests that PfPSH2 is a dual helicase, which acts on both DNA and RNA substrates. These findings suggest that this protein can be localized in both nucleus as well as cytoplasm. PSH2 is present in cytoplasm in merozoite, ring and schizont stage and to some extent in nucleus in trophozoite stages of intraerythrocytic development of *P*. *falciparum* 3D7 strain. Furthermore the colocalization with PfPABP, which is a cytoplasmic protein confirms the presence of PSH2 in cytoplasm^46^. PSH2 is also detectable in the mixed parasite lysate of *P*. *falciparum* 3D7 strain. Previous reports have suggested that PfPSH3 is nucleocytoplasmic protein and it colocalizes with PfDOZI in the cytoplasm. PfPSH2 shows similar localization as PfPSH3 and both are cytoplasmic proteins except PfPSH2 is expressed in all the intraerythrocytic developmental stages where as PfPSH3 is not expressed in ring stage. On the other hand, PfUvrD shows schizont stage specific expression and only nuclear localization in comparison to PfPSH3 and PfPSH2^36,37^. The intraerythrocytic cycle transcriptome (3D7) available on PlasmodB database (https://www.plasmodb.org) also shows that PfPSH2 is expressed in all the intraerythrocytic stages of parasite. Its transcript level starts increasing from 7 hours post invasion and peaks after 27–35 hours post invasion and then decreases slightly around 32–40 hours.

To determine the importance of PfPSH2 for the malaria parasite, dsRNA mediated growth inhibition assay was performed. PfPSH2 specific dsRNA treated parasite showed abnormal morphology and death whereas GFP dsRNA treated parasite was normal and less parasite death was observed. Recently published study has revealed the essential and non-essential genes present in the malaria parasite genome^57^. The presence of PfPSH2 (PF3D7_120200) in the list of non-mutable genes further strengthens our observation that PfPSH2 might be crucial for the survival of parasite. Similar studies were reported previously where PfUvrD dsRNA treatment to the parasite culture lead to growth defects and morphological abnormalities^58^. The semi quantitative real time PCR results suggest that the mRNA expression level of PfPSH2 gene is reduced in PfPSH2 dsRNA treated culture. Therefore, the effect on health and growth of parasite might be due to reduction in expression level of PfPSH2 mRNA. Although due to the absence of classical machinery of RNAi in *P*. *falciparum*, the mechanism of action of dsRNA is not fully known and there are several reports of inhibition of parasite growth due to antisense RNA effect^59–62^. The results of String analysis showed that majority of interacting proteins are involved in the pathways which are responsible for RNA regulation and processing. Most RNA regulation and processing occur in the cytoplasm and the presence of PfPSH2 in the cytoplasm was further strengthened by the in-silico prediction of protein-protein interactions. The unique properties of PfPSH2 such as its dual and bipolar nature of unwinding and its nucleocytoplasmic localization suggest that most likely it is a multifunctional protein involved in various aspects of nucleic acid metabolism in the parasite. The characterization of PfPSH2 will help to understand the nucleic acid metabolic pathways in the parasite. The essentiality of PfPSH2 reported in this study sets the foundation for future development of PfPSH2 as drug target.

## Methods

### In-silico analysis

PSHs in *P*. *falciparum* 3D7 strain were previously identified by the in-silico analysis^34^. PlasmoDb (release 37) database was used to retrieve the amino acid sequence of PfPSH2 (PF3D7_1202000). Schematic diagram for PfPSH2 was generated using Prosite (http://prosite.expasy.org/prosite.html)^40^. The sequences of orthologues of PfPSH2 were obtained from NCBI and multiple sequence alignments were done using Clustal Omega software (http://www.ebi.ac.uk/Tools/msa/clustalo/)^39,63^.

### Parasite blood stage culture

To culture *P*. *falciparum* 3D7 strain *in vitro* O+ human erythrocytes (4% hematocrit) in RPMI media (1640) (Invitrogen Corporation, USA), 50 mg/L hypoxanthine (Sigma Aldrich Co., USA), 0.5 g/L Albumax I (Gibco, Thermofisher Scientific Inc., USA) and 2 g/L sodium bicarbonate (Sigma Aldrich Co., USA) was used^64^. For the synchronization of parasite stages, 5% sorbitol was used to lyse parasite at stages other than rings^65^.

### Site-directed mutagenesis

The mutation was introduced in the conserved motif of the full-length PfPSH2 to observe its effect on the ATPase and helicase activity. The signature motif I’s (GTGK/ET) conserved residue lysine (K) was substituted with glutamic acid E, at amino acid position 203 (K203E). The template used for the generation of mutant PfPSH2 was full-length PfPSH2-pET28a clone and the site-directed mutagenesis (SDM) was performed by the Stratagene lightening change SDM kit according to the manufacturer’s protocol using the primers described in Supplementary table 1.

### Expression and purification of recombinant proteins

For overexpression of PfPSH2-pET28a+ clone was transformed into *E*. *coli* strain BL21 codon Plus. Overnight primary inoculation was done and secondary culture was inoculated with 2% of overnight grown primary inoculum. The secondary culture was grown up to 0.6 OD at 37 °C then 1 mM IPTG was added to induce the culture which was further allowed to grow at 16 °C for 24 hours in terrific broth. Lysis buffer of pH 7.5 (50 mM Tris–HCl, 500 mM NaCl, 0.05% Tween 20, 0.1% Triton 100) and the protease inhibitor cocktail from Roche (Sigma, St. Louis, MO, USA) was used for suspending the pellet. The sonication was done for lysis of cells and the lysate was centrifuged at 10000 rpm for 30 min. The protein in the supernatant was subjected to binding with Ni-NTA (Qiagen, GmbH, Germany) equilibrated in binding buffer (50 mM Tris–HCl pH 7.5, 500 mM NaCl, 10 mM imidazole) and protease inhibitor cocktail for 4 hours at 4 °C. The recombinant his-tagged protein was eluted with varying concentration of imidazole (50–500 mM) in chilled elution buffer (50 mM Tris–HCl pH 7.5, 500 mM NaCl, 10% (v/v) glycerol and protease inhibitor cocktail). SDS-PAGE coupled western blot analysis was performed for checking the purity of PfPSH2. For western blot analysis, anti his-tagged antibodies conjugated with horseradish peroxidase (Sigma, St. Louis, MO, USA) were used for detection. The purification of PfPSH2N and PfPSH2M were done using similar procedure.

### Ethics statement

The animal studies described in this study were approved by the ICGEB Institutional Animal Ethics Committee (IAEC Reference No. 53–3). ICGEB is licensed to conduct animal studies for research purposes under the registration number 18/1999/CPCSEA (dated 10/1/99). This is to further state that all experiments were performed in accordance with relevant guidelines and regulations.

### Polyclonal antibody generation against PfPSH2N protein

The purified fraction of PfPSH2N protein was used for the generation of polyclonal sera in the rabbit. To increase the antigenicity of the protein it was mixed with equal amount of the Freund’s complete adjuvant. For primary immunization of the rabbit 200 μg of purified protein was used and for the booster’s mixture of incomplete Freund’s adjuvant and protein was used. The bleed was taken every week after the booster immunization and the titer of sera against PfPSH2 was checked using ELISA.

### ATPase assay

To detect ATPase activity, [γ-^32^P] ATP was used and the activity was analyzed by measuring the released Pi. For ATPase assay purified PfPSH2 protein was used with ATPase buffer (20 mM Tris–HCl, pH 8.0, 8 mM DTT, 1.0 mM MgCl_2_, 20 mM KCl and 16 μg/ml BSA) and a mixture of 1 µl [γ-^32^P] ATP (~17 nM) and 1 mM cold ATP and was incubated at 37 °C for 1 hour or various time intervals. 50 ng of M13 mp19 ssDNA was added to the reaction to measure DNA-dependent ATPase activity, and the reaction mixture without protein was used as a control. To measure RNA dependent ATPase activity 50 ng of total RNA isolated from trophozoite stage of *P*. *falciparum* 3D7 strain was used. After incubation the reaction was stopped on ice and then 1 μl of reaction mix from each reaction was spotted onto thin layer chromatography (TLC) plate (Sigma). TLC buffer (0.5 M LiCl and 1 M formic acid) was used for separating hydrolyzed Pi using TLC and the plate was air dried and then scanned on a phosphoimager. All the quantitation was done using Image j software^66^.

### Preparation of DNA helicase substrate and direction-specific substrates

Helicase assay substrate and direction-specific substrates were prepared according to the protocol described (Supplementary Fig. 3)^37^.

### Preparation of RNA helicase substrate

To prepare the partially duplex RNA substrate for RNA helicase assay, 13 mer (5′-AUAGCCUCAACCG-3′) and 39 mer oligoribonucleotide (5′- GGGAGAAAUCACUCGGUUGAGGCUAUCCGUAAAGCACGC-3′) were synthesized from Sigma Aldrich. For labelling 50 ng 13 mer oligo at 5′-end, T4 polynucleotide kinase (PNK) (5U) (New England Biolabs) and 1.85 MBq of [γ-^32^P] ATP was used and reaction was incubated at 37 °C for 90 minutes and then annealed with 500 ng of 39 mer oligo by standard annealing procedure. The non-hybridized oligoribonucleotide was removed using Sepharose 4B column chromatography. The hybridized fractions were checked on 15% TBE PAGE and the selected hybridized fractions were used for RNA helicase assay.

### DNA and RNA helicase assay

The helicase assay is used to measure the unwinding activity of the protein using a partially duplex labelled substrate. The reaction mixture for DNA helicase assay (10 µl) contained purified protein, helicase buffer (20 mM Tris–HCl (pH 8.0), 8 mM DTT, 1.0 mM MgCl_2_, 1.0 mM ATP, 10 mM KCl, 4% (w/v) sucrose, 80 µg/ml BSA), ^32^P-labelled helicase substrate and incubated at 37 °C for 60 min or the time specified. The reaction was stopped by the addition of helicase dye (0.3% SDS, 10 mM EDTA, 10% Ficoll and 0.03% bromophenol blue). The substrate and unwound products were separated using a 12% non-denaturing polyacrylamide gel electrophoresis. Finally, the gel was exposed for autoradiography. All the quantitation was done using Image j software^66^. The RNA helicase assay was performed using similar method as DNA helicase assay but the duplex RNA substrate was used.

### Immunofluorescence assay

To investigate the localization and colocalization of PSH2 with PABP in the parasite, smears of parasitized red blood cells of different developmental stages were prepared on a slide and fixed using chilled methanol for 15 min at −80 °C. The slides were incubated for blocking in 10% bovine serum albumin (BSA) in phosphate buffered saline (1X PBS) in a humid chamber at 37 °C for 2 hours. The slides were washed with PBS and incubated with anti-PfPSH2N antibodies (raised in rabbit) at 1:500 dilution and PABP antibodies (raised in mice) at 1:50 dilution in PBS containing 3% BSA for 2 hours at 37 °C. The slides were then washed two times with PBST (PBS, 0.5% Tween 20) and once with PBS for 5 min each and then incubated for 1 hour at 37 °C with secondary antibody (fluorescein isothiocyanate-conjugated anti-rabbit and anti-mouse IgG (Alexa 488 and Alexa 594 respectively) diluted 1:500 in PBS containing 3% BSA). Confocal images were captured using a Bio-Rad 2100 laser-scanning microscope attached to a Nikon TE 2000U microscope and for the preparation of NIS elements Advance research software package was used (NIKON). To analyze pixel-pixel overlap of two fluorescence antibodies Pearson’s correlation coefficient provide a statistical solution for fluorescence colocalization^67^. Western blot analysis of parasite lysate was done using the method described (Supplementary Fig. 5).

### Parasite growth assay in presence of dsRNA

To analyze the effect of dsRNA, the synchronized parasite cultures with 2% hematocrit and 1% infected red blood cells (RBCs) were used. The dsRNA preparation and purification has been described in Supplementary Fig. 6A,B. The synchronized parasite culture was washed twice with incomplete RPMI medium and 20 μg dsRNA per ml was added to the culture and the mixture was dispensed in 96-well plates and kept for incubation at 37 °C. After 24 hours of incubation at 37 °C blood smears were prepared on slide and was subjected to Giemsa stain for microscopic examination of parasitemia.

### cDNA synthesis and semi quantitative real time PCR

Total RNA from Trizol (Invitrogen) treated parasite cultures was extracted using RNeasy mini kit according to manufacturer’s protocol. Isolated RNA was quantified using nano-drop (Thermo Scientific). 1 µg RNA from each experimental set of parasite culture was used to synthesize cDNA using the superscript III single strand cDNA synthesis kit according to manufacturer’s protocol. These synthesized cDNAs were used for real time PCR reactions setup and Fast SYBR Green qPCR master mix (Applied Biosystems, Life technology) was used to perform RT experiment. Real time PCR data was analyzed using ΔΔCt method. Primer details are given in Supplementary Table 1.

### String analysis

To analyze the protein-protein interactions of PfPSH2 in silico, StringDb version 10.5 online available software suite was used (http://www.string-db.org/)^47,48^. For the highly stringent predictions cut off score of 0.9 was set and then analysis was done. The analysis of the highest scoring 10 interactions was selected and represented.

## Supplementary information

Supplementary Information
