# Supplementary material for: Plasmodium falciparum specific helicase 2 is a dual, bipolar helicase and is crucial for parasite growth
Source: Sci Rep. 2019 Feb 6;9:1519. doi: 10.1038/s41598-018-38032-1 (PMC6365506; doi:10.1038/s41598-018-38032-1)
Supplement: Supplementary file 1 — Supplementary Information [file 41598_2018_38032_MOESM1_ESM.pdf]

Plasmodium falciparum **specific helicase 2 is a dual, bipolar helicase and is crucial for parasite growth**

Manish Chauhan and Renu Tuteja*

Parasite Biology Group, ICGEB, P. O. Box 10504, Aruna Asaf Ali Marg, New Delhi-110067

*To whom correspondence should be addressed

Tel. +91-11-26741358; Fax: +91-11-26742316;

Email: renu@icgeb.res.in; renututeja@gmail.com

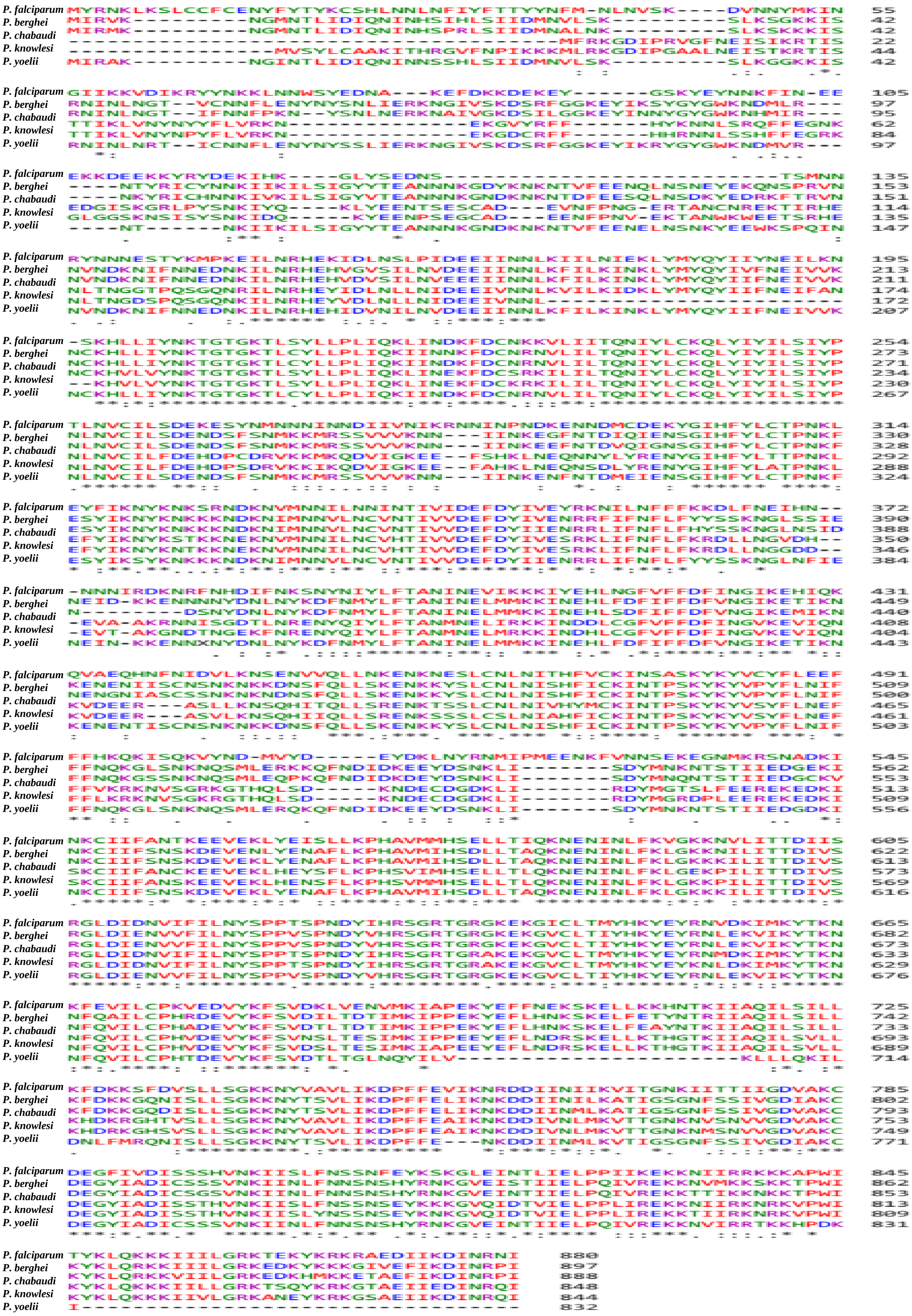

Supplementary Fig. 1A

Multiple sequence alignment of PfPSH2 with the homologs from other *Plasmodium* species

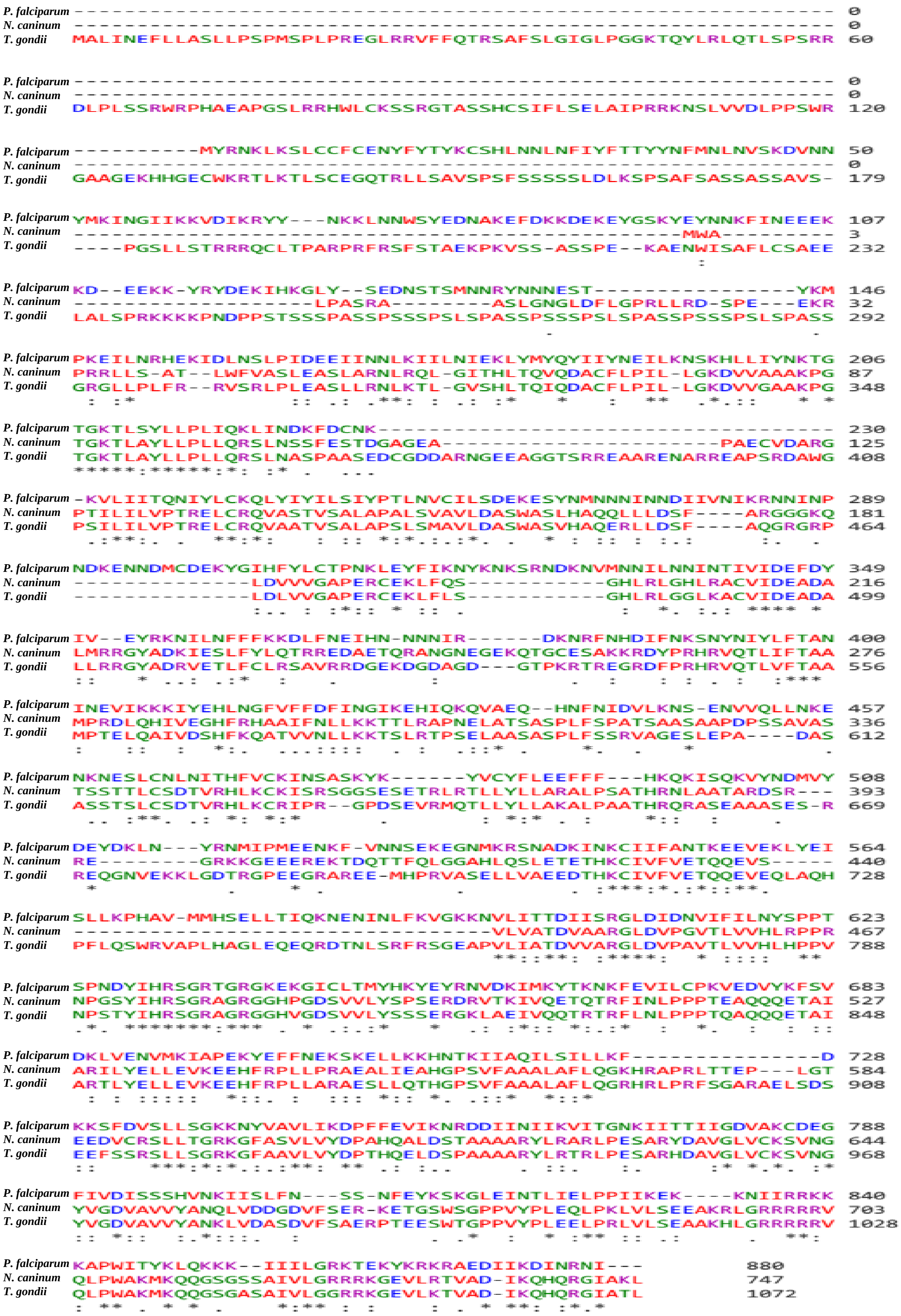

Supplementary Fig. 1B

Multiple sequence alignment of PfPSH2 with the ampicomplexan orthologs

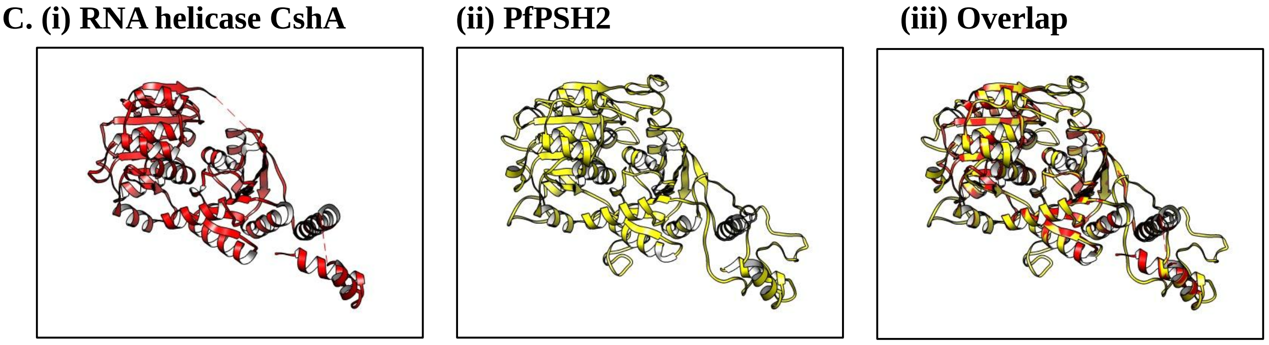

Supplementary Fig. 1C

(C) Homology modeling of PfPSH2 (i) Template for homology modelling, ATP dependent DEAD Box helicase CshA **(ii)** Predicted PfPSH2 protein model **(iii)** Overlapping model of template and PfPSH2

A. Cloning of full-length and N terminal fragment of PfPSH2. PfPSH2N is 537 nucleotides long and it encodes for 179 amino acids long protein of ~28 kDa. Both PfPSH2N and full length PfPSH2 were amplified using gene-specific primers (Supplementary Table 1). The template used for the PfPSH2N was genomic DNA because this region has no intron. Full-length PfPSH2 was amplified using cDNA as a template because PfPSH2 contains introns in its C-terminal region. To amplify PfPSH2N and full length PfPSH2; PCR was done using high fidelity DNA polymerase. Temperature gradient optimization was done in case of full length PfPSH2 and the amplification of template was achieved at annealing temperature of 58.8 °C for 1 minute and 30 seconds. Both the PCR amplified fragments were subsequently cloned in pJET 1.2 (Thermo Scientific) and were sequenced. The sequenced clones were further sub-cloned into expression vector pET28a (Novagen) and transformed into BL21 codon plus *E. coli* cells for overexpression of the protein.

Accession number for PfPSH2N is MH550160; for PfPSH2 is KX355628 and for PfPSH2M is MH046872.

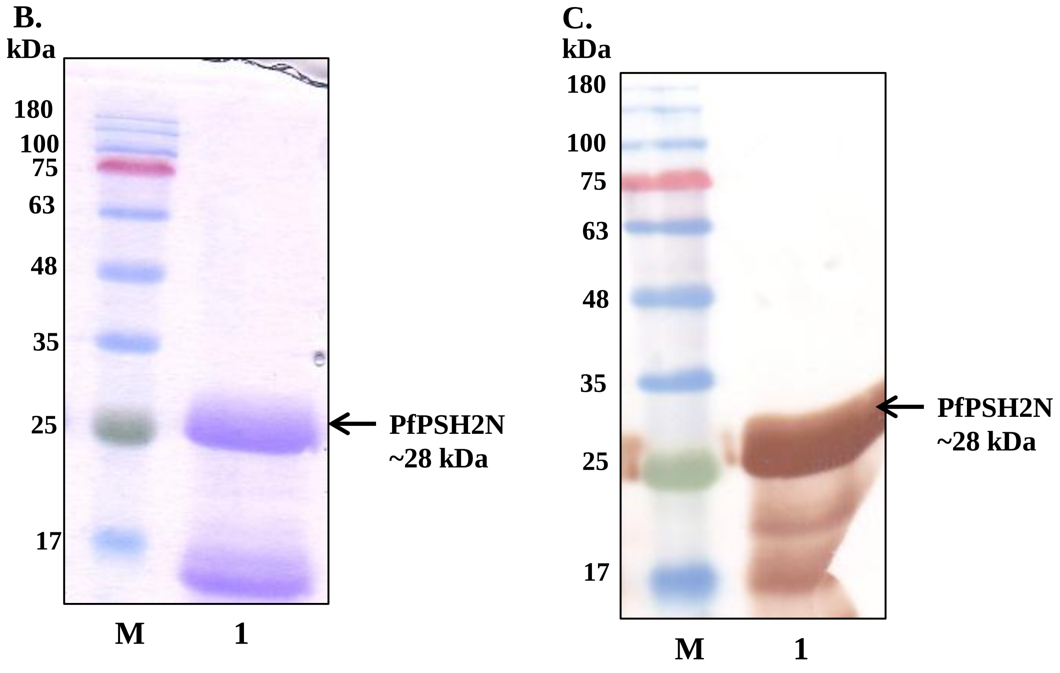

Supplementary Fig. 2

(A) Cloning of full-length and N terminal fragment of PfPSH2; (**B)** Commassie blue stained gel. Lane M is molecular weight marker and lane 1 is purified PfPSH2N protein (~28 kDa); **(C)** Western Blot analysis. Lane numbers are similar to B.

D.

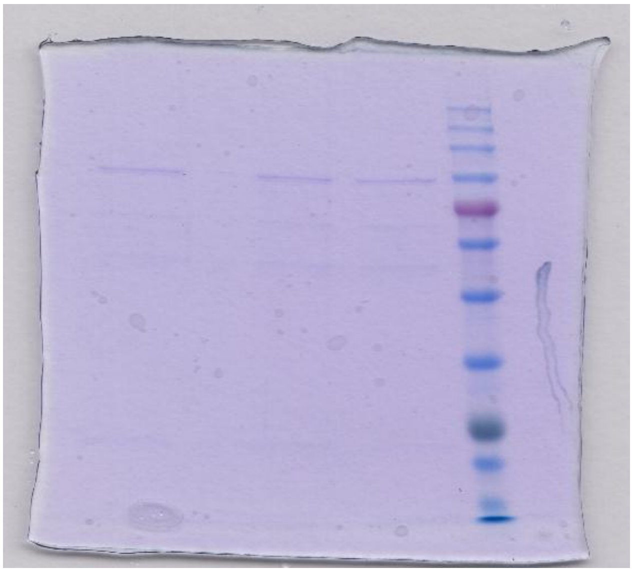

E.

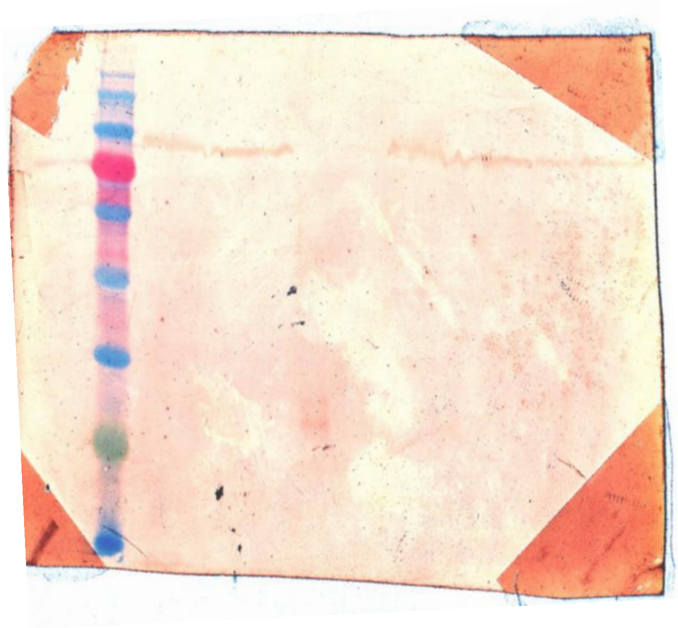

Supplementary Fig. 2

(D) Uncropped SDS gel image of PfPSH2; **(E)** Uncropped Western blot for PfPSH2;

Preparation of DNA helicase substrate and direction-specific substrates.

To analyze the ability of PfPSH2 to unwind DNA duplex; the standard strand displacement assay using the partially duplex substrate was used. For preparation of the substrate; the oligo containing 15 base-pairs of the non-complementary region $(T)_{15}$ at both the 5' and 3' ends 5'- $(T)_{15}$GTTTTCCCAGTCACGAC$(T)_{15}$-3' was synthesized. Using T4 polynucleotide kinase (PNK) (5U) (New England Biolabs) and 1.85 MBq of [γ-^{32}P] ATP (specific activity 222 TBq/mmol) the oligo was labeled at 5' end by incubating for 90 minutes at 37ºC. This labelled oligo was annealed with 0.5 μg of single-stranded circular M13mp19 (+) phage DNA using standard annealing buffer (20 mM Tris-HCl, pH 7.5, 10 mM $MgCl_2$, 100 mM NaCl, 1 mM DTT) by heating it at 95 ºC for 1 min and then allowed to cool slowly to room temperature. To remove non-hybridized oligos Sepharose 4B column (Pharmacia, Sweden) was used. The eluted fractions of the substrate were checked on a nondenaturing 12% PAGE by electrophoresis and then purified fractions were stored and used for helicase assay.

The synthesized oligodeoxynucleotide 32-mer (5′-TTCGAGCTCGGTACCCGGGGATCCTCTAGAGT-3′) was used for the preparation of the 5′ to 3′ direction-specific substrate. It was first annealed to M13mp19 ssDNA and then labelling at the 3′-OH end was done with 50 μCurie (α-^{32}P) dCTP and 5 units of DNA polymerase I (large fragment) at 23 ºC for 40 min. After labelling annealed substrate was digested with SmaI enzyme and purified as described above. For preparation of 3′ to 5′ direction-specific substrate first 5′-end labeling of 32-mer oligodeoxynucleotide was done and then it was annealed with M13mp19 ssDNA. Then labelled and annealed substrate was digested with SmaI and purified by gel filtration with the help of Sepharose 4B.

Supplementary Fig. 3

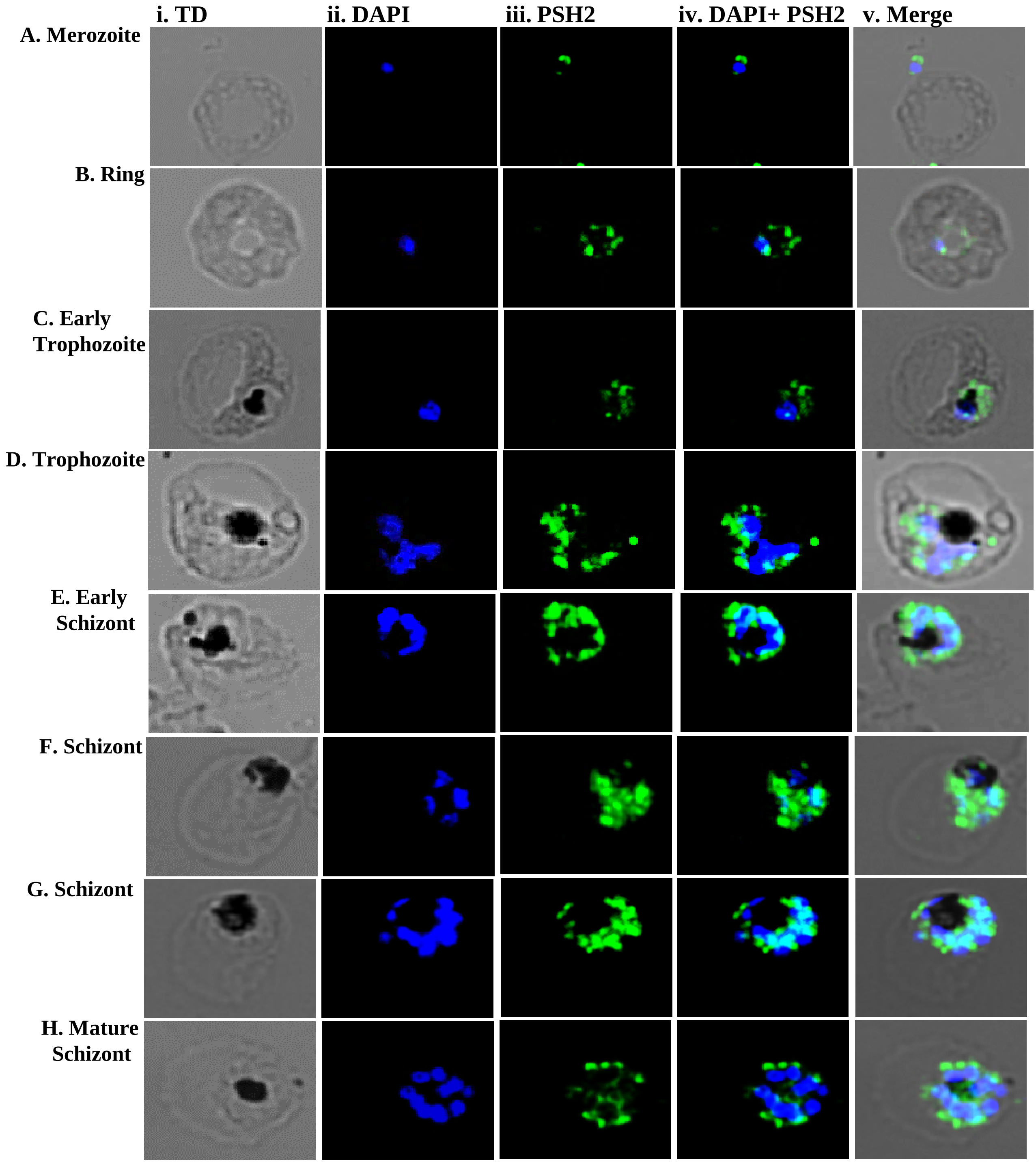

Supplementary Fig. 4

Staining with PfPSH2N sera (A) Merozoite stage, (B) Ring stage, (C) Trophozoite stage, (D) Schizont stage, (E) Early schizont stage, (F and G) Schizont stage, (H) Mature schizont stage (i) phase contrast (TD) image; (ii) image of cell stained with DAPI (blue); (iii) immunofluorescent PSH2 stained cell; (iv) DAPI + PSH2; (v) All merged

Western blot analysis of parasite lysate

The *P. falciparum* infected RBCs (10-15 % parasitemia) were treated with 0.15% saponin to lyse the RBCs and to obtain the parasite. The parasites were then lysed with lysis buffer of the kit (Pierce IP cross link kit) supplemented with 1X protease inhibitor cocktail (Roche). The complete lysis of parasite was achieved with repeated cycles of snap freeze (in liquid nitrogen) and quick thaw at 37˚C. The parasite lysate was then centrifuged at 13000 rpm for 20 minutes to separate supernatant from debris and this supernatant was used for the experiment. The anti-PfPSH2N rabbit antibodies and the pre-immune rabbit sera were cross linked to Protein A/G sepharose beads according to the protocol (Pierce IP cross link kit). The lysate was incubated with the crosslinked antibodies on orbital shaker at 4˚C for 12 hours. The mixtures were then washed and eluted with low pH elution buffer provided with the kit. The SDS PAGE and western blot analysis was performed with the eluted immunoprecipitates and the blot was probed with anti-PfPSH2N mice and pre-immune mice sera. The blot was probed with HRP-conjugated secondary antibodies (anti-mice, Sigma) and developed with DAB-peroxide (Diaminobenzidine) staining.

Supplementary Fig. 5

A.

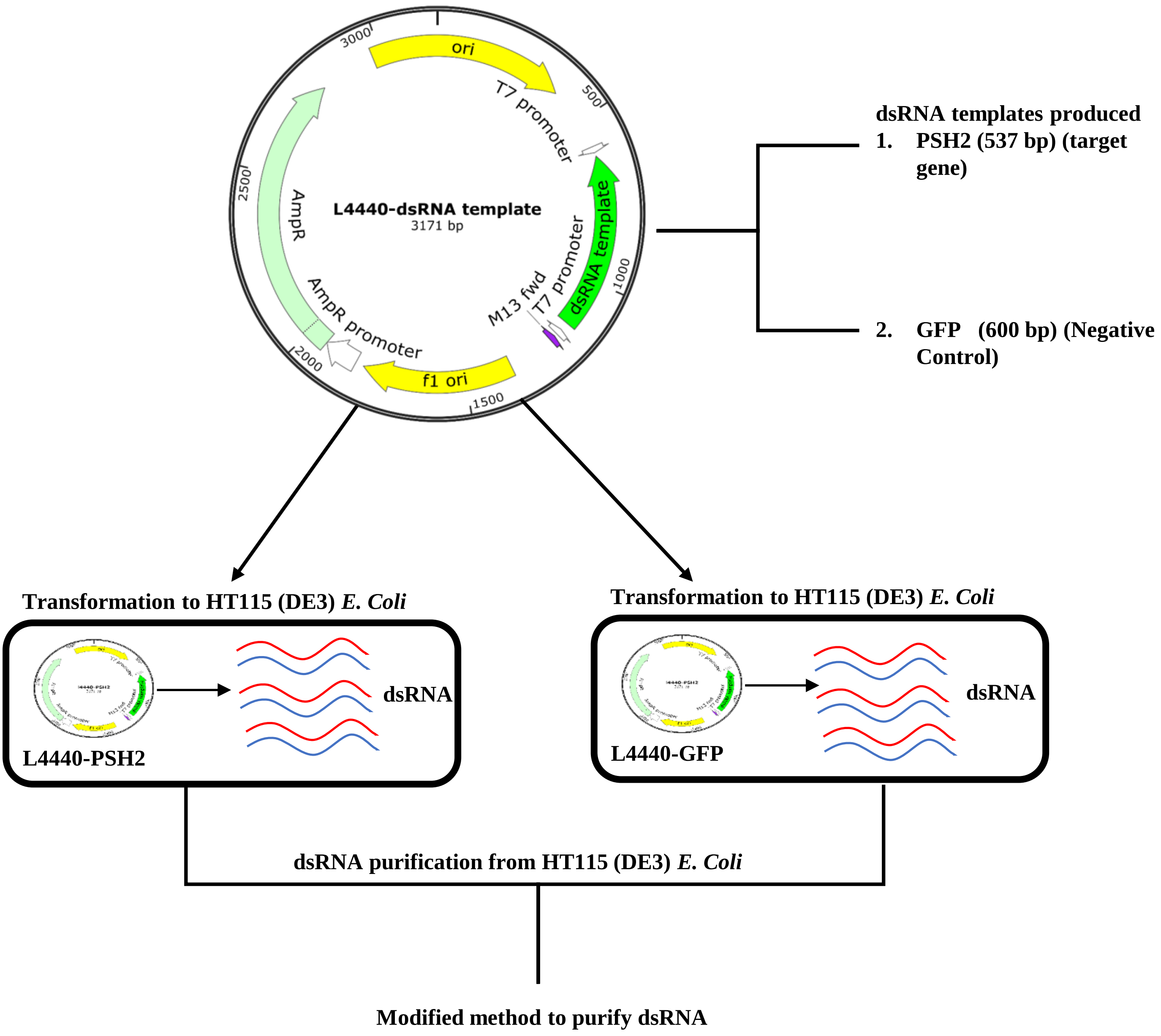

Supplementary Fig. 6

A. Cloning strategy of PfPSH2N and GFP in L4440 vector and transformation in HT115(DE3) cells

B. dsRNA Preparation and purification.

The 537-base pair PfPSH2N was amplified for cloning in to L4440 Plasmid using the specific set of primers. The amplified PCR product was sub-cloned into L4440 vector using BglII and KpnI sites present in multiple cloning site of the vector. L4440 was a gift from Andrew Fire (Addgene plasmid # 1654)[69]. Recombinant L4440 plasmid carrying PfPSH2N fragment was then transformed into HT115 (DE3) cells. For transformation of PfPSH2N/L4440 clone specially engineered RNase III deficient bacterial strain HT115 (DE3) was used. Single colony was selected from transformed HT115 (DE3) LB plate and was inoculated in LB at 37°C with shaking at 160 rpm overnight. Next morning culture was diluted 50-fold in 200 ml LB and was supplemented with 200 μg/ml ampicillin plus 12.5 μg/ml tetracycline and cultured at 37°C to OD600=1. Bacterial culture was induced with 2 mM IPTG and the bacteria were incubated with shaking for an additional 4 hours at 37°C. HT115 (DE3) cells were obtained from University of Minnesota *C. elegan*s RNAi Center. To purify dsRNA from HT115 (DE3) cells, bacterial culture was pelleted at 13000 rpm for 2 minutes. The cell pellet was resuspended in 1/20 of the initial volume of 1M ammonium acetate and 10 mM EDTA and an equal volume of phenol:chloroform:isoamyl alcohol (125:24:1). To lyse the bacteria, samples were boiled at 65°C for 20 minutes and centrifuged at 12000 rpm for 15 minutes[69]. The clear upper phase was collected and mixed with equal volume of chloroform:isoamyl alcohol (24:1) and vortexed for 10 seconds and incubated at room temperature for 5 minutes and centrifuged at 12000 rpm for 15 minutes. The clear upper phase was collected in fresh DNase/RNase free centrifuge tube and an equal volume of isopropanol was added and incubated at -20°C for 30 minutes, then centrifuged at 12000 rpm for 20 minutes. The pellet was washed twice with 70% ethanol and air dried. Tris EDTA pH 7.5 was added to resuspend the pellet. DNaseI and RNaseA treatment were given to remove DNA and ssRNA contaminations.

Supplementary Fig. 6

A.

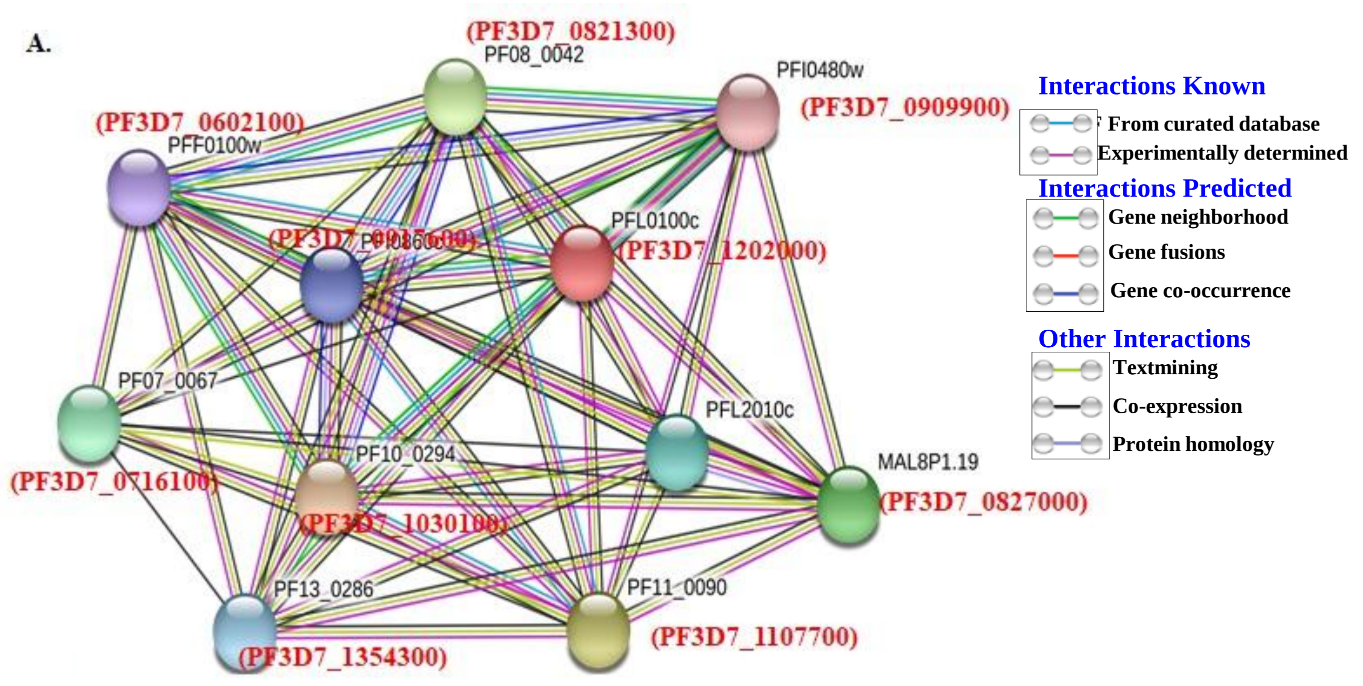

B. Details of the predicted interacting partners of PfPSH2

S.NO	Plasmodb Id		Name	Pathway	Score
	Old	New			
1.	PF10_0294	PF3D7_1030100	PRP22 putative	Pre mRNA splicing	0.989
2.	PF11_0090	PF3D7_1107700	pescadillo homolog	Ribosomal RNA maturation	0.972
3.	PF08_0042	PF3D7_0821300	DHX36, putative	mRNA regulation	0.947
4.	MAL8P1.19	PF3D7_0827000	DBP10, putative	60s ribosomal subunit biogenesis	0.945
5.	PF07_0067	PF3D7_0716100	protein SDA1, putative	60s ribosomal export	0.943
6.	PFL2010c	PF3D7_1241800	DBP9, putative	Ribosomal biogenesis	0.927
7.	PF13_0286	PF3D7_1354300	large subunit rRNA methyltransferase, putative	Methylation	0.913
8.	PFI0860c	PF3D7_0917600	PRP43, putative	Pre mRNA splicing	0.907
9.	PFF0100w	PF3D7_0602100	ATP-dependent RNA helicase, putative	NA	0.906
10.	PFI0480w	PF3D7_0909900	helicase SKI2W, putative	Exosome mediated RNA decay	0.897

Supplementary Fig. 7

In silico protein-protein interaction analysis. Protein-protein interaction prediction was done using String DB version 10.5 **(A)** StringDb output image of interaction and side panel displays the type of interaction predicted; **(B)** Predicted interacting partners and the pathways in which they are involved.

Supplementary Table 1. List of primers

S.No	Primer Name	Sequence	Restriction site
1.	PSH2 Forward	*TTACATATGATGTATAGAAATAAATTGAAGAG*	NdeI
2.	PSH2 Reverse	*TTACTCGAGTTCTATATTTAAAATAATCTTAAGGT*	XhoI
3.	PSH2N Forward	*TTACATATGATGTATAGAAATAAATTGAAGAG*	NdeI
4.	PSH2N Reverse	*TTA CTCGAG TTCTATATTTAAAATAATCTTAAGGT*	XhoI
5.	mutPFPSH2-F	*AAAACAGGTACAGGG GAA ACCTTAAGTTATT*	NA
6.	mutPFPSH2-R	*AATAACTTAAGGTTT CCC CTGTACCTGTTTT*	NA
7.	dsRNAPSH2-F	*TTA AGATCT ATGTATAGAAATAAATTGAAGAG*	BglII
8.	dsRNAPSH2-R	*TTA GGTACC TTCTATATTTAAAATAATCTTAAGGT*	KpnI
	**RT-Primers**	**sequence**	**Restriction site**
9.	PSH2 Forward	*TGAGTATAAGAGTAAAGGATTAGAA*	NA
10.	PSH2 Reverse	*ATATCCTCAGCTCGTTTTCTTTTG*	NA
11.	18s Forward	*GTTAAGGGAGTGAAGACGATCAGA*	NA
12.	18s Reverse	*AACCCAAAGACTTTGATTTCTCATAA*	NA
